# A Nationwide Study on the Impact of Routine Testing for *EGFR* Mutations in Advanced NSCLC Reveals Distinct Survival Patterns Based on *EGFR* Mutation Subclasses

**DOI:** 10.3390/cancers13143641

**Published:** 2021-07-20

**Authors:** Bart Koopman, Betzabel N. Cajiao Garcia, Chantal C. H. J. Kuijpers, Ronald A. M. Damhuis, Anthonie J. van der Wekken, Harry J. M. Groen, Ed Schuuring, Stefan M. Willems, Léon C. van Kempen

**Affiliations:** 1 Department of Pathology and Medical Biology, University of Groningen, University Medical Center Groningen, P.O. Box 30.001, 9700 RB Groningen, The Netherlands; b.koopman@umcg.nl (B.K.); b.n.cajiao.garcia@umcg.nl (B.N.C.G.); e.schuuring@umcg.nl (E.S.); s.m.willems@umcg.nl (S.M.W.); 2 Foundation PALGA, De Bouw 123, 3991 SZ Houten, The Netherlands; Chantal.Epskamp-Kuijpers@palga.nl; 3 Netherlands Comprehensive Cancer Organisation (IKNL), P.O. Box 19079, 3501 DB Utrecht, The Netherlands; R.Damhuis@iknl.nl; 4 Department of Pulmonary Diseases, University of Groningen, University Medical Center Groningen, P.O. Box 30.001, 9700 RB Groningen, The Netherlands; a.j.van.der.wekken@umcg.nl (A.J.v.d.W.); h.j.m.groen@umcg.nl (H.J.M.G.)

**Keywords:** *EGFR*, non-small cell lung cancer, molecular diagnostics, nationwide, real-world, survival

## Abstract

**Simple Summary:**

The presence of an *EGFR* activating mutation in tumors of non-small-cell lung cancer patients enables effective targeted therapy towards EGFR. Studies that describe a nationwide uptake of *EGFR* testing, the impact of the switch from single-gene *EGFR* to multi-gene testing, and the clinical response towards EGFR inhibitors in first-line treatment are limited. From 2013 to 2017 the percentage of patients routinely tested for *EGFR* mutations increased from 73% to 81% in the Netherlands. A strong shift towards *EGFR* testing as part of a multi-gene next generation sequencing analysis was observed. However, this did not change the percentage of *EGFR* mutations that were reported for this patient population, which remained stable at 12%. When treated with EGFR inhibitors that were available in a routine clinical setting prior to 2018, clear differences were observed between the type of *EGFR* mutation and survival.

**Abstract:**

*EGFR* mutation analysis in non-small-cell lung cancer (NSCLC) patients is currently standard-of-care. We determined the uptake of *EGFR* testing, test results and survival of *EGFR*-mutant NSCLC patients in the Netherlands, with the overall objective to characterize the landscape of clinically actionable *EGFR* mutations and determine the role and clinical relevance of uncommon and composite *EGFR* mutations. Non-squamous NSCLC patients diagnosed in 2013, 2015 and 2017 were identified in the Netherlands Cancer Registry (NCR) and matched to the Dutch Pathology Registry (PALGA). Overall, 10,254 patients were included. Between 2013–2017, the uptake of *EGFR* testing gradually increased from 72.7% to 80.9% (*p* < 0.001). Multi-gene testing via next-generation sequencing (increased from 7.8% to 78.7% (*p* < 0.001), but did not affect the number of detected *EGFR* mutations (*n* = 925; 11.7%; 95% confidence interval (CI), 11.0–12.4) nor the distribution of variants. For patients treated with first-line EGFR inhibitors (*n* = 651), exon 19 deletions were associated with longer OS than L858R (HR 1.58; 95% CI, 1.30–1.92; *p* < 0.001) or uncommon, actionable variants (HR 2.13; 95% CI, 1.60–2.84; *p* < 0.001). Interestingly, OS for patients with L858R was similar to those with uncommon, actionable variants (HR 1.31; 95% CI, 0.98–1.75; *p* = 0.069). Our analysis indicates that grouping exon 19 deletions and L858R into one class of ‘common’ *EGFR* mutations in a clinical trial may mask the true activity of an EGFR inhibitor towards specific mutations.

## 1. Introduction

Non-small cell lung cancer (NSCLC) is the leading cause of global cancer-related deaths [1]. Activating mutations in *EGFR* account for the second most common mechanism of malignant transformation in NSCLC in western Europe and the United States [2]. Clinical trials have demonstrated that first-line treatment with an EGFR tyrosine kinase inhibitor (TKI), such as gefitinib [3], erlotinib [4], afatinib [5], dacomitinib [6], or osimertinib [7]. improves survival of advanced NSCLC patients with *EGFR* exon 19 deletions and L858R point mutations. Therefore, molecular diagnostics to detect these and other *EGFR* mutations has been standard-of-care in Europe since 2010 [8,9,10]. Molecular testing has since evolved from a single-gene polymerase chain reaction-based approach to the implementation of multiplex analyses such as next-generation sequencing (NGS) in routine diagnostics for the detection of multiple gene variants in a limited amount of tissue [11,12]. A multiplex analysis is particularly useful for NSCLC due to often limited amount of available tissue and the increasing number of predictive markers beyond *EGFR* (including *BRAF*, *ERBB2* and *MET*) [13,14,15]. The Dutch National Healthcare Institute does not specify the methodology or commercially available companion diagnostic test that must be used for the evaluation of these biomarkers. Instead, individual molecular laboratories perform local validation of sequencing techniques according to national accreditation guidelines (ISO-NEN-15189:2012 since 2016/2017) [16]. All NGS panels used in the Netherlands cover the full region of interest in *EGFR* (exons 18–21).

There have been limited evaluations of the real-world, population-level effect of changes in routine *EGFR* testing and the introduction of multiplex testing on the landscape of *EGFR* mutations and overall survival rates. Furthermore, the clinical relevance of uncommon and composite *EGFR* mutations remains elusive. In the Netherlands, all patients diagnosed with cancer are registered in the Netherlands Cancer Registry (NCR), managed by the Netherlands Comprehensive Cancer Organization (IKNL). In addition, the nationwide network and registry of histo- and cytopathology in the Netherlands (PALGA) maintains a database of all pathology reports from pathology departments in the Netherlands [17]. The effects of routine *EGFR* mutation testing for advanced NSCLC patients on a population-level scale in 2013, 2015 and 2017 were evaluated following a query of both registries. During this period, only gefitinib, erlotinib and afatinib were available for use in first-line treatment.

## 2. Materials and Methods

### 2.1. Patient Selection

Testing for *EGFR* mutations has been standard-of-care in the Netherlands since 2011 [18], and NGS has gradually been implemented into the routine setting for non-squamous NSCLC (ns-NSCLC). Therefore, data were requested from the NCR and PALGA for advanced NSCLC patients diagnosed in 2013, 2015 and 2017. All patients recorded in the NCR who were diagnosed with advanced adenocarcinoma of the lung, adenosquamous carcinoma of the lung and NSCLC not otherwise specified (NOS) were included. Patients were matched with the nationwide PALGA registry to retrieve the pathology reports. Data requests were approved by the scientific and privacy committees of IKNL (application numbers K15.115, K16.264, and K18.311) and PALGA (application numbers LZV1172, LZV2016-91, LZV2018-199).

### 2.2. Data Extraction and Handling

Variables retrieved from the NCR included sex, age at diagnosis, morphology code (ICD-O 3rd edition), type of first-line treatment (categories including chemotherapy, radiotherapy, surgery, targeted therapy, other therapy, a combination of these, or no therapy), overall survival (OS) (time in days from diagnosis to death or data cut-off) and vital status as of 1 February 2020. Molecular testing results were manually extracted from the pathology reports by dedicated researchers (CCHJK, BNCG, BK). Extracted variables included whether testing was performed or not, the type of molecular test(s) used to detect *EGFR* mutations, an identifier of the pathology department that requested the test, and the reported *EGFR* mutation status. The type of molecular test(s) used were corrected to exclude secondary testing on new tissue obtained at progressive disease.

**Figure 1:**
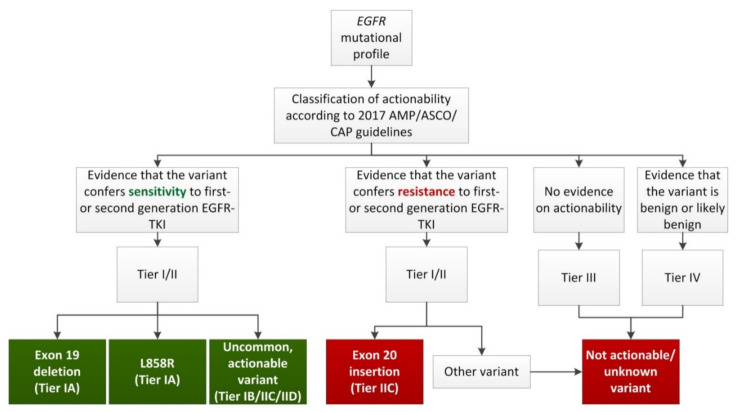
Flowchart used for classification of *EGFR* mutational profiles. Grouping of *EGFR* mutational profiles included in this study, based on the frequency of the mutation(s) and the corresponding AMP/ASCO/CAP level of evidence for actionability with first- or second-generation EGFR-TKI. Groups in green (Exon 19 deletion, L858R and Uncommon, actionable variants) are considered sensitive to first- or second-generation EGFR-TKI, groups in red (Exon 20 insertion and Not actionable/unknown variants) are considered resistant to first- or second-generation EGFR-TKI. In case of a double variant, actionability was assessed for the combination. However, if evidence for the combination was lacking, the patient was assigned according to the highest tier of the individual variants. Abbreviations: AMP, Association for Molecular Pathology; ASCO, American Society of Clinical Oncology; CAP, College of American Pathologists; *EGFR*, epidermal growth factor receptor gene; TKI, tyrosine kinase inhibitor.

Reported *EGFR* driver mutations were reannotated in accordance with the Human Genome Variant Society recommendations for the description of sequence variants [19]. Each variant was classified for predicted actionability with EGFR-TKI as ‘sensitive’ or ‘no benefit’, with level of evidence tiers based on the 2017 Association for Molecular Pathology (AMP)/American Society of Clinical Oncology (ASCO)/College of American Pathologists (AMP) guidelines [20]. Patients with *EGFR* mutations were grouped into one of five nominal categories based on the frequency of the reported *EGFR* mutations and the AMP/ASCO/CAP level of evidence (Figure 1). These categories included: exon 19 deletion (sensitive, Tier IA); L858R (sensitive, Tier IA); uncommon, actionable (sensitive, Tier IB/IIC/IID); exon 20 insertion (no benefit expected, Tier IIC) or not actionable/unknown (no benefit expected, Tier I–IV, except for exon 20 insertions). In case of a double variant, actionability was assessed for the combination. For example, the combination of uncommon, actionable variants E709A and G719A has been reported to be sensitive to afatinib [21], and the combination is thus tiered IIC (uncommon, actionable). However, if evidence for the combination was lacking, the patient was assigned according to the highest Tier of the individual variants. For instance, patients with a combination of an exon 19 deletion (Tier IA) and a variant of unknown significance (Tier III) were assigned to category ‘exon 19 deletion’. Classification was performed by a certified clinical scientist in molecular pathology (LCvK) [22], and only considered for first- and second-generation EGFR-TKI (gefitinib, erlotinib, afatinib) due to the unavailability of first-line osimertinib treatment in the study period. Clinical data processing was performed in accordance with the General Data Protection Regulation (EU) 2016/679.

### 2.3. Statistical Analysis

Statistical analysis was performed with SPSS version 23 (SPSS Inc., Chicago, IL, USA). Comparisons were performed for patient characteristics, testing rates and *EGFR* mutation detection rates between the three years of diagnosis (2013, 2015 and 2017) and between testing modalities (multi-gene assays or single-gene tests). Multi-gene assays were defined as NGS technologies or massARRAY. Proportional differences between years were assessed using Pearson’s Chi-square analysis, and, if significant, a subgroup analysis was performed with Fisher’s exact tests. Differences between testing modalities were assessed using Fisher’s exact tests.

Median OS from date of diagnosis was estimated with the Kaplan-Meier method, including 95% confidence intervals (CI), and tested for significance with the Log-rank test. For *EGFR*-mutant patients treated with first-line targeted therapy, uni- and multivariate Cox regression analyses were performed to correct for the co-variables age, sex, year of diagnosis and tumor histology. Variables with a *p*-value < 0.05 in the univariate analysis were included in the multivariate analysis (forward stepwise logistic regression). Hazard ratio (HR) and 95% CI were calculated. Differences were considered statistically significant at a *p*-value < 0.05.

## 3. Results

### 3.1. Patients Included in the Analysis

**Figure 2:**
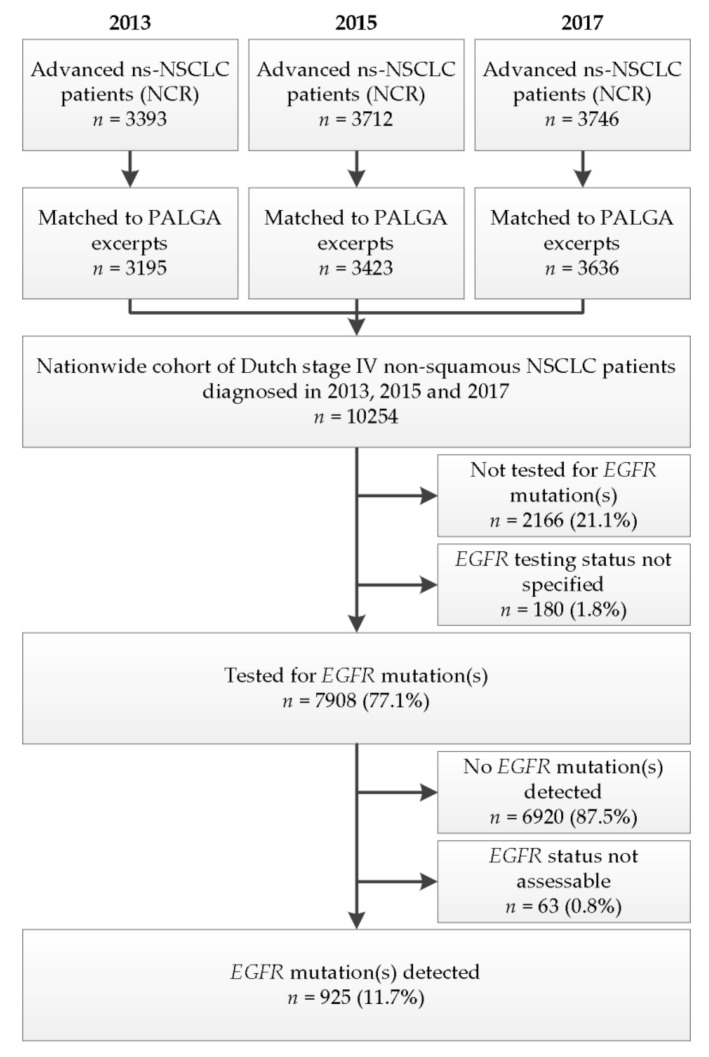
Patient selection. Flowchart depicting the selection of patients for inclusion in this study. Abbreviations: *EGFR*, epidermal growth factor receptor gene; PALGA, Dutch Pathology Registry; NCR, Netherlands Cancer Registry; ns-NSCLC, non-squamous non-small cell lung cancer.

The NCR recorded 3393, 3712 and 3746 advanced ns-NSCLC patients in 2013, 2015 and 2017, respectively. Of these, 3195 (2013), 3423 (2015) and 3636 (2017) could be matched to excerpts in the PALGA registry, creating a population-level cohort of 10,254 Dutch advanced ns-NSCLC patients (Figure 2). Patient characteristics are shown in Table 1. The mean age for the total population was 66.9 years. A small majority of patients was male (53.6%). The majority of patients were diagnosed with adenocarcinoma (86.3%), followed by NSCLC-not otherwise specified (NSCLC-NOS) (13.1%), and adenosquamous carcinoma (0.6%).

### 3.2. EGFR Testing Rates, Inter-Pathology Department Variance and Assays Used

**Figure 3:**
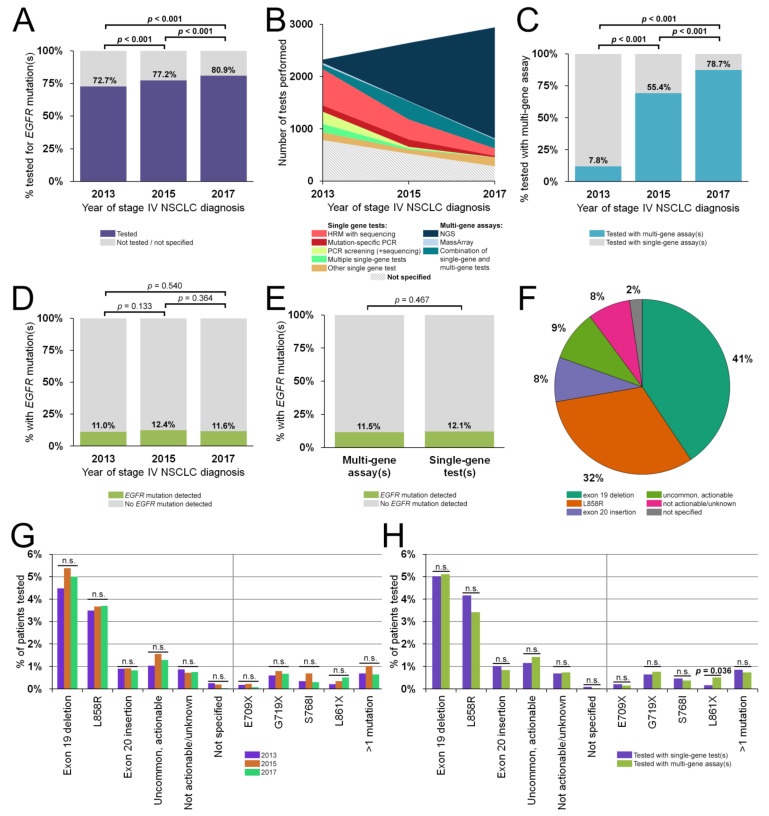
*EGFR* testing rates, methods and *EGFR* mutations. (**A**) Percentage of patients tested for *EGFR* mutations, by year of diagnosis. (**B**) Distribution of *EGFR* testing methods in number of tests by year. (**C**) Percentage of patients tested with single-gene versus multi-gene assays, by year of diagnosis. (**D**,**E**) Percentage of patients tested for *EGFR* mutations that tested positive for one or more *EGFR* mutation(s), by year of diagnosis (**D**) and multi-gene assay versus single-gene assay (**E**). (**F**) Distribution of *EGFR* mutation classes. (**G**,**H**) Comparison of distributions of *EGFR* mutation classes and occurrence of specific uncommon mutations, by year of diagnosis (**G**) and single-gene assay versus multi-gene assay (**H**). Abbreviations: *EGFR*, epidermal growth factor receptor gene; HRM, high-resolution melting; NGS, next-generation sequencing; n.s., not significant; NSCLC, non-small cell lung cancer; PCR, polymerase chain reaction.

Out of the total population of 10,254 advanced NSCLC patients, 77.1% (95% CI, 76.3–77.9%; *n* = 7908) were tested for *EGFR* mutations. The testing rate increased significantly throughout the study period, from 72.7% (95% CI, 71.2–74.2%) in 2013 to 77.2% (95% CI, 75.8–78.6%) in 2015 (*p* < 0.001), up to 80.9% (95% CI, 79.6–82.2%) in 2017 (*p* < 0.001) (Figure 3A). A commonly reported reason for not performing an *EGFR* mutation analysis was the lack of sufficient suitable tissue or a low percentage of tumor cells. However, reasons for not performing an *EGFR* mutation analysis were not systematically captured in the pathology reports used in our study. Therefore, it could not be determined with high confidence which other technical, patient or infrastructural factors contributed to not testing for *EGFR* mutations. 

Samples from the patients in this study originated from 52 different pathology departments in the Netherlands. Forty-one departments reported *EGFR* mutation test results for 10 or more patients in each of the three time periods (2013, 2015 and 2017). The majority of departments improved their testing rates and the inter-pathology department variance in requesting *EGFR* testing gradually decreased from 2013 to 2017 (Figure S1). In 2017, only four out of the 41 departments (9.8%) underperformed compared to the national average. In other words, these departments reported fewer *EGFR* mutation tests (<60% of the eligible patients) than expected for their respective tumor volumes (based on the lower 99.7% CI limit) and the national average of 80.9% in 2017.

The landscape of tests used to detect *EGFR* mutations changed in the study years. Whereas the most common tests in 2013 were high-resolution melting (HRM), mutation-specific polymerase chain reaction (PCR), single-gene sequencing (such as Sanger sequencing) or combinations thereof, *EGFR* testing was mostly performed (78.7%) with multi-gene assays in 2017 (Figure 3B). The use of multi-gene assays as first-line molecular diagnostics surged from 7.8% in 2013 to 55.4% in 2015 (*p* < 0.001), and further increased to 78.7% in 2017 (*p* < 0.001) (Figure 3C). Laboratories generally use custom or generic NGS panels validated in accordance with national accreditation guidelines (ISO-NEN-15189:2012 since 2016/2017). Examples of more common NGS assays are custom AmpliSeq-panels on an IonTorrent platform [23], the TruSeq Amplicon Cancer Panel-based NGS using a MiSeq Personal sequencer [24], and the nationally aligned single-molecule Molecular Inversion Probe (smMIP) PATHv2D panel on an Illumina platform [25]. The panels used by Dutch molecular pathology laboratories all covered the full region of interest in *EGFR* (exons 18–21).

### 3.3. Landscape of EGFR Mutations in Untreated Dutch NSCLC Patients

Of the 7908 patients tested for *EGFR* mutations at initial diagnosis, one or more mutations were reported in 11.7% of all cases (95% CI, 11.0–12.4%; *n* = 925) (Table 2). Female patients were more likely to harbor *EGFR* mutations (16.0%; 95% CI, 14.8–17.2%; *n* = 600) than male patients (7.8%; 95% CI, 7.0–8.6; *n* = 325; *p* < 0.001). There were no significant differences in *EGFR* positivity between the three study years (Figure 3D) nor between the use of single-gene or multi-gene assays (Figure 3E). The majority of patients harbored a classical, actionable exon 19 deletion (41% of 925 cases) or L858R (32%), whereas uncommon but actionable mutations (9%), exon 20 insertions (8%), and other not actionable/unknown variants (8%) constituted a much smaller proportion of the *EGFR*-mutant patients (Figure 3F). On a variant class- or single mutant-level, no significant distribution differences were observed for the three study years (Figure 3G) nor for the use of single-gene or multi-gene assays (Figure 3H), except for *EGFR* L861X. This variant (either alone or in combination with a second *EGFR* variant) was slightly more common in patients tested with a multi-gene assay (*n* = 20; 0.5% of those tested for *EGFR* mutations) compared to those tested with single-gene tests (*n* = 5; 0.2%; *p* = 0.036).

### 3.4. Impact of EGFR Testing on First-Line Targeted Therapy and Subsequent Overall Survival

Treatment and survival data were available for 10,237 out of 10,254 patients (99.8%). This included 390 patients with an exon 19 deletion, 287 patients with L858R, 103 patients with an uncommon, actionable variant, 69 patients with an exon 20 insertion, 61 patients with a not actionable/unknown variant and 6972 patients without *EGFR* mutation. Patients who were not tested for *EGFR* mutations (*n* = 2343) were excluded from the analysis, as were patients reported to be *EGFR* mutation positive but for whom the exact variant was not reported (and could thus not be classified) (*n* = 12).

#### 3.4.1. Overall Survival of *EGFR*-Mutant Patients Versus Those without *EGFR* Mutations

**Figure 4:**
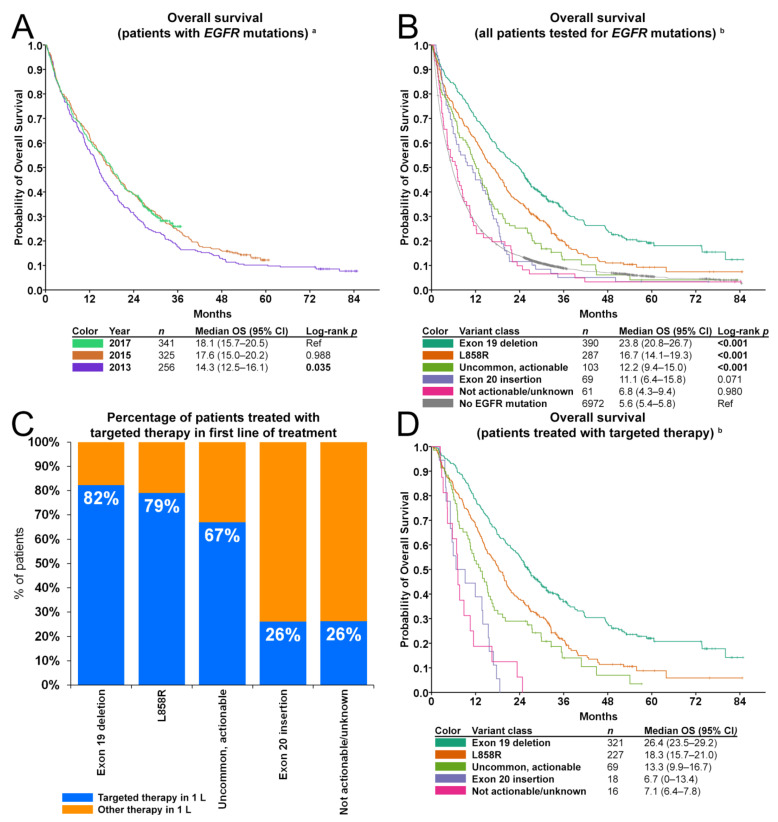
First-line targeted therapy and overall survival. (**A**) Kaplan-Meier curve depicting overall survival by year of diagnosis, irrespective of first-line treatment. Censored cases are indicated with a +. (**B**) Kaplan-Meier curve depicting overall survival by mutation class, irrespective of first-line treatment. Censored cases are indicated with a +. (**C**) Percentage of patients treated with targeted therapy in first-line of treatment, by *EGFR* mutation class. (**D**) Kaplan-Meier curve depicting overall survival of *EGFR*-mutant patients treated with targeted therapy in first-line of treatment. Censored cases are indicated with a +. ^a^ Excluding patients with missing follow-up data (*n* = 3). ^b^ Excluding patients with missing follow-up data (*n* = 3) and patients reported to have a non-specified *EGFR* mutation (*n* = 12). Abbreviations: 1 L; first-line of treatment; CI, confidence interval; *EGFR*, epidermal growth factor receptor gene; p, level of significance; OS, overall survival.

OS in patients with any *EGFR* mutation was higher for those diagnosed in 2017 (median 18.1 months; 95% CI, 15.7–20.5) compared to 2013 (median 14.3 months; 95% CI, 12.5–16.1; *p* = 0.035), but similar to 2015 (median 17.6 months; 95% CI, 15.0–20.2; *p* = 0.988) (Figure 4A). Irrespective of first-line treatment, distinct survival patterns were observed in the different *EGFR* mutation subclasses (Figure 4B). Median OS in patients without *EGFR* mutations was 5.6 months (95% CI, 5.4–5.8) in the period 2013–2017. Evaluation of OS for the various *EGFR* mutation classes revealed a favorable OS for patients with exon 19 deletions (23.8 months; 95% CI, 20.8–26.7; *p* < 0.001), L858R (16.7 months; 95% CI, 14.1–19.3; *p* < 0.001) and uncommon, actionable variants (12.2 months; 95% CI, 9.4–15.0; *p* < 0.001). In contrast, median OS of patients with exon 20 insertions (11.1 months; 95% CI, 6.4–15.8; *p* = 0.071) or with not actionable/unknown variants (6.8 months; 95% CI, 4.3–9.4; *p* = 0.980) was comparable to the OS of patients without an *EGFR* mutation.

#### 3.4.2. Overall Survival in Patients Treated with First-Line Targeted Therapy

Out of the 910 patients with *EGFR* mutation(s) and available follow-up, 651 (72%) received first-line treatment with targeted therapy. Of note, this included patients in the not actionable/unknown group (Tier III–IV), which today would likely not be treated with targeted therapy. The proportion receiving targeted therapy was not affected by year of diagnosis (70%, 70% and 74% in 2013, 2015 and 2017, respectively; *p* = 0.332) (Figure S2). There was no information on which inhibitor was used. However, in 2013–2017, gefitinib, erlotinib and afatinib were the only EGFR inhibitors used in first-line therapy [26]. The remaining patients (representing 29% of all *EGFR*-mutated patients) were either treated with a non-targeted modality (*n* = 171; 19%) or did not receive treatment (*n* = 88; 10%). The NCR did not register reasons for not treating patients with targeted therapy, nor was this information available in the pathology reports. Therefore, reasons for not treating patients with targeted therapy could not be investigated.

The proportion of patients receiving first-line targeted therapy was highest for those with an exon 19 deletion (321/390; 82%) or L858R mutation (227/287; 79%), lower for those with uncommon, actionable variants (69/103; 67%) and smallest for those with exon 20 insertions (18/69; 26%) and not actionable/unknown variants (16/61; 26%) (Figure 4C). These proportions were not affected by year of diagnosis (Figure S2). Distinct differences in survival were observed between the predefined categories of *EGFR* mutations (Figure 4D). Median OS in patients treated with first-line targeted therapy was 26.4 months for patients with exon 19 deletions (95% CI, 23.5–29.2), 18.3 months for patients with L858R (95% CI, 15.7–21.0), 13.3 months for patients with uncommon, actionable variants (95% CI, 9.9–16.7), 6.7 months for patients with exon 20 insertions (95% CI, 0–13.4), and 7.1 months for patients with not actionable/unknown variants (95% CI, 6.4–7.8). 

Uni- and multivariable Cox regression analyses were performed to evaluate whether the type of *EGFR* mutation is associated with OS (Table 3). Because OS for patients with *EGFR* exon 20 insertions and patients with not actionable/unknown variants treated with first- and second-generation EGFR inhibitors was comparably poor (Figure 4D), these groups were pooled into one category dubbed as “resistant/unknown variants”. A higher age (*p* < 0.001), male sex (*p* < 0.008) and diagnosis in 2013 (*p* = 0.011) were significantly associated with worse outcome in the univariate analyses. When corrected for these factors, patients with *EGFR* exon 19 deletions showed superior outcomes compared to those with L858R (hazard ratio (HR) 1.6; 95% CI, 1.3–1.9; *p* < 0.001), uncommon, actionable variants (HR 2.1; 95% CI, 1.5–2.7; *p* < 0.001) and resistant/unknown variants (HR 4.7; 95% CI, 3.2–6.8; *p* < 0.001). OS of patients with *EGFR* L858R when corrected for age, sex and year of diagnosis was not different from patients with uncommon actionable *EGFR* mutations (HR 1.31; 95% CI, 1.0–1.8; *p* = 0.069). Furthermore, patients with resistant/unknown variants had a worse outcome compared to those with *EGFR* L858R (HR 3.0; 95% CI, 2.1–4.3; *p* < 0.001) or those with uncommon actionable variants (HR 2.4; 95% CI, 1.6–3.6; *p* < 0.001).

In 2017, the third-generation EGFR inhibitor osimertinib was approved for use in patients who developed the *EGFR* T790M mutation as a mechanism of resistance. In this year, of the 254 patients who received an EGFR inhibitor, 27 (11%) developed the T790M within one year after diagnosis. An additional 16 patients (6%) developed this mutation in the remainder of 2018. The T790M mutation was detected on the background of an exon 19 deletion (27 out of 321 (8.4%) treated with targeted therapy), the L858R mutation (15/227; 6.6%) and the composite G719S/L861Q mutation (*n* = 1). Because the reports were limited to 2018, the number of long-term responders cannot be estimated. The NCR data did not contain information about the type of therapy in second-line nor the duration of response to second-line treatment. Therefore, a survival analysis to investigate the impact of resistance-targeted treatment options could not be performed. There is insufficient data to investigate whether the treatment-induced T790M is more prevalent on the background of an exon 19 deletion than L858R.

## 4. Discussion

In this study, the impact of routine testing for *EGFR* mutations was investigated in the Dutch population of patients with newly diagnosed advanced ns-NSCLC in 2013, 2015 and 2017. This analysis revealed that, despite a significant increase in testing rate and a nationwide shift from single-gene testing to multi-gene assays such as NGS, the *EGFR* positivity rate and distributions of specific (common and uncommon) alterations that can be observed at primary diagnosis remained comparable. In addition, the analysis of real-world treatment outcome data in the Netherlands revealed distinct survival patterns for patients with different classes of *EGFR* mutations when treated in first-line with first- (gefitinib, erlotinib) or second-generation (afatinib) EGFR-TKI. The results of this population-based analysis hold not only value for policy makers and healthcare providers, but also offer insight into the frequency and consequences of reported uncommon and composite variants for clinicians and scientists.

### 4.1. Impact of Routine EGFR Testings on Detection of EGFR Mutations and Overall Survival

Testing for *EGFR* mutations at primary diagnosis has been standard-of-care for advanced NSCLC patients in the Netherlands since 2011 [9,27]. Our analysis showed that the testing rate has since then gradually but significantly increased from 72.7% in 2013 to 80.2% in 2017 (*p* < 0.001). Whether nationwide testing rates further increased thereafter will be the subject of future studies. Reasons for not performing an *EGFR* mutation analysis were not systematically captured in the pathology reports. The availability and suitability of sufficient tissue, patient factors negating the need for testing (death prior to testing, poor condition, or personal preference provided at shared decision-making visits) are likely important factors. However, it cannot be excluded that a percentage of eligible NSCLC patients are not offered predictive testing despite the availability of tissue and increasing number of registered (EGFR-)targeted therapies. 

The prevalence of *EGFR* mutations in the Dutch population of ns-NSCLC is 11.7% (95% CI, 11.0–12.4%). This was comparable to the prevalence found in a smaller Dutch cohort in 2012 (10.6% in adenocarcinoma of the lung) [28], though slightly lower than previously reported in a pooled prevalence analysis of studies with European subjects (14.1%) [29]. As expected, *EGFR* mutations associated with EGFR inhibitor-induced on-target resistance mutations were not observed in this untreated cohort, except for one case. This single case of *EGFR* T790M without another mutation might suggests a germline event, but cannot be concluded without additional testing. 

The uptake of NGS as the method of choice for *EGFR* analysis (from 8% in 2013 to 79% in 2017) did not impact the nationwide detection rate of *EGFR* mutations nor the distribution of uncommon mutations. This is likely because the majority of single-gene tests in this study were sequencing techniques (such as pyrosequencing, Sanger sequencing or HRM in combination with sequencing) that covered the full region of interest in *EGFR* (exons 18–21). The only variant impacted by the shift to multi-gene testing was the uncommon *EGFR* L861X mutation in exon 21. This variant was slightly more common in patients tested with a multi-gene assay compared to single-gene tests (*p* = 0.036). The first reports on the actionability of this variant with first-generation EGFR-TKI emerged in 2018 [30]. It is conceivable that this continuously increasing knowledge resulted in a more frequent reporting of previously unrecognized uncommon yet actionable *EGFR* variants. In addition, single-gene tests available in 2013—such as cobas^®^ EGFR Mutation Test v1 (Roche Holding AG, Basel, Switzerland)—might not have been designed to capture an L861X mutation due to its previously unknown clinical significance. As such, the introduction of *EGFR* mutation analysis by NGS likely contributed to the discovery of treatment options for the current uncommon *EGFR* mutations. Our results indicate that the utilization of NGS did not adversely impact the detection rate for *EGFR* mutations underscoring the accurate detection of mutations. 

Median OS in the Dutch population of advanced ns-NSCLC patients with *EGFR* mutations was 18.1 months in 2017, which is comparable to other real-world studies with European subjects [31]. Although median OS in patients diagnosed in 2015 was comparable (17.6 months), patients diagnosed in 2013 survived shorter (14.3 months, *p* = 0.035). This may be because patients diagnosed in 2013 generally did not have access to second-line testing and novel treatment options. For example, a multitude of potential resistance mechanisms to first- and second-generation EGFR-TKIs therapies have been reported, most commonly the T790M resistance mutation [32]. The activity of third-generation inhibitors, such as osimertinib, in first and later lines of therapy, is unaffected by this mutation [33]. In addition, checkpoint inhibition has been approved for patients with tumors expressing PD-L1. This has improved survival rates in advanced NSCLC patients [34]. Checkpoint inhibitors are not used in first- or second-line treatment of *EGFR*-mutant patients as they do not improve OS in these patients [35]. Unfortunately, assessment of the impact of the introduction of the new treatment modalities could not be performed as the NCR database lacks information on second and later lines of treatment.

### 4.2. Beyond EGFR

The number of options for targeted therapy for NSCLC patients beyond EGFR has strongly increased. Different therapies are now registered or being tested in trials for patients with an activating mutation *ALK* [36], *BRAF* [13], *ERBB2* [15], *MET* [14], *NTRK1-3* [37], *RET* [38], and *ROS1* [39]. In addition, trials with specific KRAS G12C inhibitors demonstrate strong clinical benefit with registration expected by the end of 2021 [40]. As such, the panel of therapeutically relevant biomarkers in NSCLC is continuously increasing. Testing patients for all these predictive markers using consecutive single-gene tests is inefficient considering the often limited amount of available tissue. Therefore, NGS has become standard-of-care in the Netherlands to simultaneously test for all relevant actionable mutations also beyond *EGFR* as an efficient diagnostic work-up [25]. Although the content of the panels has changed since 2017, the current core set of genes relevant for NSCLC was already analyzed in 2017. Due to higher volumes of NGS tests, the turnaround times and costs per sample have decreased. Improvements of library preparation methods and optimization of sample procurement has decreased the number of samples that could not be analyzed due to low tissue volume.

Publicly available cohort databases such as the AACR project GENIE database indicate that *EGFR*-activating mutations are mutually exclusive with other known driver mutations [2]. However, co-occurrence of mutations in other genes may affect survival and response to EGFR-TKI [41]. Notably, the prognostic value of *TP53* mutations in combination has been demonstrated [42]. Patients with concurrent *EGFR* and *TP53* mutations may benefit from combining EGFR-TKI with anti-VEGF therapies such as bevacizumab [43], ramucirumab [44], or apatinib [45]. Because *TP53* was not part of the list of genes that must be sequenced in the context of NSCLC in the Netherlands in the study period [18], most laboratories did not report mutations in these genes. As such, our results could not be stratified for co-occurrence of *EGFR* and *TP53* mutations.

### 4.3. A Clinical Evidence-Driven Reclassification of EGFR Mutations

Previous reports on nationwide real-world data regarding the effect of *EGFR* mutation status and response to EGFR inhibitors generally lack detailed information with respect to *EGFR* mutation type [26,31,46,47,48]. In smaller cohorts, *EGFR* mutation status and response rates have been reported, but irrespective of evidence of response to first- and second-generation EGFR-TKI, often including T790M resistance mutations, exon 20 insertions and variants of unknown significance [49,50,51,52,53,54]. Furthermore, the *EGFR* L858R mutation and exon 19 deletions are often pooled together as ‘common’ mutations [53,55], due to their frequency but despite a difference in response in the Asian population [56]. In a recent, unpublished study, Robichaux et al. suggested four subgroups: classical-like, T790M-like, exon 20 insertions and ATP binding-pocket volume-reducing (PVR) mutations [57]. However, also in this study exon 19 deletions and L858R were pooled into the classical-like group, and included combinations between a common and an uncommon mutation in the ‘PVR’ group. In contrast, it may be more appropriate to classify variants based on their respective sensitivity to individual inhibitors in terms of survival [58]. Thus, we differentiated *EGFR* variants in terms of frequency and actionability with first- and second-generation EGFR-TKI. The classification scheme proposed by the 2017 AMP/ASCO/CAP guidelines [20], was used to individually classify all detected variants into *EGFR* “exon 19 deletion” (Tier I), “L858R” (Tier I) and into three groups of uncommon variants: “exon 20 insertions” (Tier II), “uncommon, actionable variants” including known double mutations such as S768I/L861Q and G719A/S768I (Tier I or II) and “not actionable/unknown variants” (Tier III or IV) (Figure 1 and Table 4). This classification scheme revealed distinct survival characteristics in patients treated with first-line targeted therapy. 

Patients with an *EGFR* exon 19 deletion, L858R or uncommon actionable mutation benefit from first- and second-generation EGFR inhibitors, albeit to a different extent (median OS 95% CI: 20.8–26.7, 14.1–19.3, 9.4–15.1 months, respectively). OS for patients with *EGFR* exon 20 insertions and not actionable/unknown variants was comparable to patients without an *EGFR* mutation who were not treated with an inhibitor. The current study confirmed the superior OS for patients with an *EGFR* exon 19 deletions compared the L858R mutation (adjusted HR of L858R versus exon 19 deletion was 1.57; *p* < 0.001) [56]. When corrected for covariates such as age, sex and year of diagnosis, the difference in OS for patients with an *EGFR* L858R or uncommon actionable mutation was not significant (HR uncommon, *EGFR* actionable variants versus L858R: 1.311; *p* = 0.069). It should be noted that the majority of these patients were treated with first-generation *EGFR*-TKIs gefitinib or erlotinib [26], which may be less effective in patients with uncommon *EGFR* mutations than afatinib [49,54], or osimertinib [77]. Afatinib was likely used in a minority of patients [26], and osimertinib was not available for first-line treatment in the study period. Thus, survival rates for L858R and uncommon, actionable variants may be even more similar with newer agents but has to be investigated. Collectively, these results reinforce the notion that future clinical trials need to avoid pooling *EGFR* L858R and exon 19 deletions into a single category, and warrants further investigation into the similarities between L858R and ‘uncommon, actionable’ variants.

Both patients with *EGFR* exon 20 insertions and patients with variants classified as ‘not actionable/unknown’ showed dismal survival when treated with first- or second-generation EGFR-TKI in first-line. Both categories were considered as ‘resistant’ to first- and second-generation EGFR-TKI: the former category because these are known to be non-responsive to first- and second-generation TKI [78], and the latter because (pre)clinical evidence on actionability is lacking. However, these perspectives might change with new evidence: for example, there are promising novel agents and combination therapies currently under investigation that may be effective against *EGFR* exon 20 insertions, including amivantamab [79], poziotinib [80], and mobocertinib [81], or combining osimertinib or afatinib with cetuximab [82]. In the Netherlands, all Tier II and III *EGFR* variants are nowadays commonly discussed in Molecular Tumor Boards [83], resulting in favorable treatment outcomes [61]. 

### 4.4. Limitations of the Study

Although the PALGA registry offers nationwide coverage on pathology testing in the Netherlands, the collected information in the study period represented narrative pathology reports in routine diagnostics. Thus, if patients were tested for *EGFR* mutations for research purposes or if the pathologist in charge did not include testing results in the pathology report, patients may have been missed and the testing rate may be underestimated.

Similarly, the NCR, which also has nationwide coverage and therefore allows for unique real-world analyses, has several limitations. First, it does not include treatment-specific information such as best overall response or progression-free survival and is limited to first-line treatment. Second, the NCR did not register reasons for not treating patients with targeted therapy; thus, we could not investigate why circa 20% of patients with common *EGFR* mutations were not treated with EGFR-targeted therapy. Finally, information on which EGFR-TKI was specifically used for each patient was not provided, but was restricted to first- (gefitinib, erlotinib) and second-generation (afatinib) inhibitors that could have been used in the period 2013–2017. In 2017, osimertinib became available which has made a positive impact on overall survival. Due to the limitations of the NCR database, this impact could not be quantified. The clinical impact of currently available third-generation inhibitors on *EGFR* variants in second-line testing and multiple lines of treatment was therefore not the subject of this study.

Despite the increase in the use of NGS, the current study did not capture mutations in genes other than *EGFR*. As such, frequencies of other driver mutations are not reported and a complete landscape of mutations in the known drivers is not presented.

## 5. Conclusions

In the period 2013–2017, *EGFR* mutation testing in the Netherlands has transformed from a single-gene approach to the nationwide implementation of NGS using a multigene panel for predictive biomarker testing including *EGFR* according to the current Dutch national guideline for lung cancer [10]. This shift did not affect the overall detection rate of *EGFR* mutations (11.7%) nor the distribution of mutations. In patients treated with first-line targeted therapy, *EGFR* exon 19 deletions, L858R, and uncommon, actionable variants were all independently associated with improved survival compared to patients with *EGFR* exon 20 insertions or not actionable/unknown variants. Furthermore, we demonstrated that overall survival in patients with L858R is more comparable to those with uncommon, actionable mutations than to those with *EGFR* exon 19 deletions. These results indicate that distinguishing between *EGFR* exon 19 deletions and L858R mutations, as well as classifying actionability of uncommon variants using the 2017 AMP/ASCO/CAP guidelines, is a more appropriate method to predict actionability and survival than the grouping of mutations into ‘common’ and ‘uncommon’ variants based on their frequencies in the patient population.

## Figures and Tables

**Table 1:** Patient characteristics, *EGFR* testing method and mutation status at initial diagnosis of advanced NSCLC.

Characteristic	Total	2013	2015	2017
Cases, *n* (%)	10,254 (100)	3195 (100)	3423 (100)	3636 (100)
Sex				
Male	5498 (53.6)	1730 (54.1)	1820 (53.2)	1947 (53.5)
Female	4757 (46.4)	1465 (45.9)	1603 (46.8)	1689 (46.5)
Age				
Mean (range)	66.9 (20–101)	66.2 (23–98)	67.0 (24–97)	67.4 (20–101)
<65 years	4133 (40.3)	1386 (43.4)	1355 (39.6)	1392 (38.3)
≥65 years	6121 (59.7)	1809 (56.6)	2068 (60.4)	2244 (61.7)
Tumor histology				
Adenocarcinoma	8845 (86.3)	2724 (85.3)	2961 (86.5)	3160 (86.9)
Adenosquamous carcinoma	62 (0.6)	19 (0.6)	25 (0.7)	18 (0.5)
NSCLC, NOS	1347 (13.1)	452 (14.1)	437 (12.8)	458 (12.6)
*EGFR* mutation testing				
Tested	7908 (77.1)	2324 (72.7)	2643 (77.2)	2941 (80.9)
Not tested ^a^	2166 (21.1)	765 (24.0)	766 (22.4)	635 (17.5)
Testing status not specified	180 (1.8)	106 (3.3)	14 (0.4)	60 (1.6)
*EGFR* test performed at initial diagnosis (% of *n* tested)				
Single-gene test	2350 (29.7)	1356 (58.3)	653 (24.7)	341 (11.6)
Fragment analysis	8 (0.1)	8 (0.3)	–	–
HRM (±sequencing)	1215 (15.4)	693 (29.8)	382 (14.5)	140 (4.8)
Immunohistochemistry	20 (0.3)	20 (0.9)	–	–
Mutation-specific PCR	290 (3.7)	121 (5.2)	141 (5.3)	28 (1.0)
PCR screening (±sequencing) ^b^	259 (3.3)	232 (10.0)	27 (1.0)	–
Pyrosequencing	109 (1.4)	2 (0.1)	12 (0.5)	95 (3.2)
Sanger sequencing	257 (3.2)	115 (4.9)	67 (2.5)	75 (2.6)
Combination of different single-gene tests	192 (2.4)	165 (7.1)	24 (0.9)	3 (0.1)
Multi-gene assay	3958 (50.1)	181 (7.8)	1463 (55.4)	2314 (78.7)
MassARRAY	59 (0.7)	35 (1.5)	8 (0.3)	16 (0.5)
NGS	3299 (41.7)	63 (2.7)	1111 (42.0)	2125 (72.3)
Combination of single- and multi-gene assays	600 (7.6)	83 (2.6)	344 (13.0)	176 (5.9)
Test not specified	1600 (20.2)	787 (33.9)	527 (19.9)	286 (9.7)
*EGFR* mutation status (% of *n* tested)				
*EGFR* mutation(s) reported	925 (11.7)	256 (11.0)	328 (12.4)	341 (11.6)
No *EGFR* mutation(s)	6920 (87.5)	2046 (88.0)	2297 (86.9)	2577 (87.6)
*EGFR* testing failed	63 (0.8)	22 (1.0)	18 (0.7)	23 (0.8)

^a^ Reasons for not performing an *EGFR* mutation analysis were not systematically captured in the pathology reports used in our study. Therefore, it could not be determined what factors (technical and/or patient-specific) contributed to not testing for *EGFR* mutations. ^b^ Diagnostic algorithm consisting of screening with mutation-specific PCR, followed by *EGFR* sequencing. Abbreviations: *EGFR*, epidermal growth factor receptor gene; HRM, high-resolution melting; NGS, next-generation sequencing; NOS, not otherwise specified; NSCLC; non-small cell lung cancer; PCR, polymerase chain reaction.

**Table 2:** *EGFR* mutations in the Dutch population of advanced non-squamous NSCLC patients by year of diagnosis (A) and molecular diagnostic modality (B).

**A**	**By Year of Advanced NSCLC Diagnosis**						
**Mutation(s)**		**Total**	**2013**	**2015**		**2017**	***p* ^a^**
Tested for Mutations, *n* (%)		7908 (100)	2324 (100)	2643 (100)		2941 (100)	
Any *EGFR* mutation		925 (11.7)	256 (11.0)	328 (12.4)		341 (11.6)	0.303
Distribution of predefined classes of sensitivity							
Exon 19 deletion		393 (5.0)	104 (4.5)	142 (5.4)		147 (5.0)	0.347
L858R		287 (3.6)	81 (3.5)	97 (3.7)		109 (3.7)	0.905
Exon 20 insertion		69 (0.9)	21 (0.9)	24 (0.9)		24 (0.8)	0.917
Uncommon, actionable		103 (1.3)	24 (1.0)	41 (1.6)		38 (1.3)	0.274
Not actionable/unknown		61 (0.8)	20 (0.9)	19 (0.7)		22 (0.7)	0.836
Not specified		12 (0.2)	6 (0.3)	5 (0.2)		1 (0.0)	0.097
Distribution of uncommon *EGFR* mutation(s)							
>1 *EGFR* mutation		62 (0.8)	16 (0.7)	27 (1.0)		19 (0.6)	0.233
E709X		12 (0.2)	4 (0.2)	6 (0.2)		2 (0.1)	0.299
G719X		55 (0.7)	14 (0.6)	21 (0.8)		20 (0.7)	0.713
S768I		35 (0.4)	8 (0.3)	18 (0.7)		9 (0.3)	0.076
L861X		29 (0.4)	5 (0.2)	9 (0.3)		15 (0.5)	0.206
**B**	**By Molecular Diagnostic Modality**						
**Mutation(s)**		**Total**	**Single-Gene Test(s)**		**Multi-Gene Assay**		***p* ^c^**
Cases, *n* (%)		6308 (100) ^b^	2350 (100)		3958 (100)		
Distribution of predefined classes of sensitivity							
Exon 19 deletion		320 (5.1)	118 (5.0)		202 (5.1)		0.906
L858R		233 (3.7)	98 (4.2)		135 (3.4)		0.129
Exon 20 insertion		57 (0.9)	24 (1.0)		33 (0.8)		0.492
Uncommon, actionable		83 (1.3)	27 (1.1)		56 (1.4)		0.424
Not actionable/unknown		45 (0.7)	16 (0.7)		29 (0.7)		0.878
Not specified		3 (0.0)	2 (0.1)		1 (0.0)		0.560
Distribution of individual uncommon *EGFR* mutation(s)							
>1 *EGFR* mutation		49 (0.8)	20 (0.9)		29 (0.7)		0.657
E709X		11 (0.2)	5 (0.2)		6 (0.2)		0.551
G719X		45 (0.7)	15 (0.6)		30 (0.8)		0.645
S768I		26 (0.4)	11 (0.5)		15 (0.4)		0.685
L861X		24 (0.4)	4 (0.2)		20 (0.5)		0.036

Values in bold indicate significant differences. ^a^ Level of significance tested with Pearson’s Chi-square test, considered significant at *p* < 0.05. ^b^ Does not include patients for whom the testing modality was not reported (*n* = 1600). ^c^ Level of significance tested with Fisher’s exact test, considered significant at *p* < 0.05. Abbreviations: *EGFR*, epidermal growth factor receptor gene; HRM, high-resolution melting; NGS, next-generation sequencing; NOS, not otherwise specified; NSCLC; non-small cell lung cancer; PCR, polymerase chain reaction.

**Table 3:** Uni- and multivariate Cox regression overall survival analyses for *EGFR* mutation-positive NSCLC patients treated with first-line targeted therapy (*n* = 651).

Factor		Univariate Analysis			Multivariate Analysis ^a^		
	*n*	HR	95% CI	*p*	HR	95% CI	*p*
**Age (cont.)**	651	1.02	1.01–1.03	**<0.001**			
**Age (in years)**							
<65 years	258	Ref	Ref	Ref			
≥65 years	393	1.26	1.06–1.51	**0.011**			
**Sex**							
Male	223	Ref	Ref	Ref			
Female	428	0.78	0.65–0.94	**0.008**			
**Tumor histology**							
Adenocarcinoma	630	Ref	Ref	Ref			
NSCLC, NOS	19	1.45	0.88–2.39	0.142			
Adenosquamous carcinoma	2	2.23	0.56–8.96	0.258			
**Year of diagnosis**							
2013	175	1.32	1.07–1.63	**0.011**			
2015	223	Ref	Ref	Ref			
2017	253	1.01	0.82–1.26	0.902			
***EGFR* mutation class (Ref: Exon 19 deletion)**							
Exon 19 deletion	321	Ref	Ref	Ref	Ref	Ref	Ref
L858R	227	1.58	1.30–1.92	**<0.001**	1.57	1.29–1.90	**<0.001**
Uncommon, actionable variant	69	2.13	1.60–2.84	**<0.001**	2.05	1.54–2.74	**<0.001**
Resistant/unknown variants ^b^	34	5.05	3.48–7.33	**<0.001**	4.67	3.21–6.80	**<0.001**
***EGFR* mutation class (Ref: L858R)**							
L858R	227	Ref	Ref	Ref	Ref	Ref	Ref
Uncommon, actionable variant	69	1.35	1.01–1.80	**0.046**	1.31	0.98–1.76	0.069
Resistant/unknown variants ^b^	34	3.19	2.19–4.63	**<0.001**	2.98	2.05–4.34	**<0.001**
***EGFR* mutation class (Ref: uncommon, actionable variant)**							
Uncommon, actionable variant	69	Ref	Ref	Ref	Ref	Ref	Ref
Resistant/unknown variants ^b^	34	2.37	1.55–3.63	**<0.001**	2.27	1.48–3.49	**<0.001**

Values in bold indicate significant differences. ^a^ Corrected for age (continuous), sex, and year of diagnosis. ^b^ Because patients with *EGFR* exon 20 insertions and patients with not actionable/unknown variants had comparably poor outcomes (Figure 3D), these groups were pooled into one category named “resistant/unknown variants”. Abbreviations: CI, confidence interval; cont., continuous variable; *EGFR*, epidermal growth factor receptor gene; HR, hazard ratio; NOS, not otherwise specified; NSCLC; non-small cell lung cancer; *p*, level of significance; Ref, reference group.

**Table 4:** Rationale for classification of expected first- and second-generation EGFR-TKI sensitivity for *EGFR* variants reported in the Dutch population in 2013, 2015 and 2017.

Variant(s)	*n* (%) ^a^	Rationale	Prediction	LoE	Group
**R108K**	1 (0.11%)	Known gain of function in brain tumors [59], but actionability unknown in NSCLC	no benefit	III	not actionable/unknown
**C595F**	1 (0.11%)	No evidence on pathogenicity or actionability	no benefit	III	not actionable/unknown
**Q701K**	1 (0.11%)	No evidence on pathogenicity or actionability	no benefit	III	not actionable/unknown
**L703F**	1 (0.11%)	No evidence on pathogenicity or actionability	no benefit	III	not actionable/unknown
**R705S**	1 (0.11%)	No evidence on pathogenicity or actionability	no benefit	III	not actionable/unknown
**E709_T710delinsD**	3 (0.32%)	Clinical sensitivity to EGFR-TKI reported [60,61]	sensitive	IID	uncommon, actionable
**E709A/G719A**	4 (0.43%)	Clinical sensitivity to EGFR-TKI reported [21]	sensitive	IIC	uncommon, actionable
**E709A/G719R**	1 (0.11%)	Considered comparable to E709A/G719A	sensitive	IIC	uncommon, actionable
**E709A/G719S**	1 (0.11%)	Considered comparable to E709A/G719A	sensitive	IIC	uncommon, actionable
**E709D**	1 (0.11%)	No evidence on pathogenicity or actionability. Similar amino acid properties between Glu (E) and Asp (D), thus no effect expected	no benefit	III	not actionable/unknown
**E709K/G719S**	1 (0.11%)	Clinical sensitivity to EGFR-TKI reported [62]	sensitive	IIC	uncommon, actionable
I715fs*	1 (0.11%)	No evidence on pathogenicity or actionability	no benefit	III	not actionable/unknown
**G719_S720delinsAF**	1 (0.11%)	Considered comparable to G719A	sensitive	IID	uncommon, actionable
**G719A**	18 (1.95%)	Clinical sensitivity to EGFR-TKI reported [63]	sensitive	IIC	uncommon, actionable
**G719A/D761Y**	1 (0.11%)	Considered comparable to G719A	sensitive	IIC	uncommon, actionable
**G719A +** **Exon 20 insertion, NOS**	1 (0.11%)	No EGFR-TKI sensitivity expected due to exon 20 insertion, grouped accordingly	no benefit	IIC	exon 20 insertion
**G719A/L861Q**	3 (0.32%)	Clinical sensitivity to EGFR-TKI reported [64]	sensitive	IIC	uncommon, actionable
**G719A/R776H**	1 (0.11%)	Considered comparable to G719A	sensitive	IIC	uncommon, actionable
**G719A/S768I**	5 (0.54%)	Clinical sensitivity to EGFR-TKI reported [65]	sensitive	IIC	uncommon, actionable
**G719A/V769M**	1 (0.11%)	Considered comparable to G719A	sensitive	IIC	uncommon, actionable
**G719C**	2 (0.22%)	Clinical sensitivity to EGFR-TKI reported [66]	sensitive	IIC	uncommon, actionable
**G719C/S768I**	6 (0.65%)	Clinical sensitivity to EGFR-TKI reported [67]	sensitive	IIC	uncommon, actionable
**G719S**	1 (0.11%)	Clinical sensitivity to EGFR-TKI reported [66]	sensitive	IIC	uncommon, actionable
**G719S/L747S**	1 (0.11%)	Considered comparable to G719S	sensitive	IIC	uncommon, actionable
**G719S/L861Q**	1 (0.11%)	Considered comparable to G719A/L861Q	sensitive	IIC	uncommon, actionable
**G719S/S768I**	1 (0.11%)	Considered comparable to G719A/S768I and G719C/S768I	sensitive	IIC	uncommon, actionable
**G719X, NOS**	2 (0.22%)	Similar grouping as other G719 substitutions	sensitive	IIC	uncommon, actionable
**G719X, NOS/S768I**	3 (0.32%)	Similar grouping as other G719X/S768I variants	sensitive	IIC	uncommon, actionable
**G724A/S768I**	1 (0.11%)	Considered comparable to G724S/S768I	sensitive	IIC	uncommon, actionable
**G724S/S768I**	1 (0.11%)	Clinical sensitivity to EGFR-TKI reported [68]	sensitive	IID	uncommon, actionable
**c.2184 + 19G > A**	1 (0.11%)	Likely a SNP due to high allele frequency in general population (3.5%; GnomAD) ^b^	no benefit	IV	not actionable/unknown
L730fs*1	1 (0.11%)	No evidence on pathogenicity or actionability	no benefit	III	not actionable/unknown
**I740_K745dup**	2 (0.22%)	Clinical sensitivity to osimertinib reported [69], but no evidence on first- or second-generation TKI	no benefit	III	not actionable/unknown
**A743S**	1 (0.11%)	No evidence on pathogenicity or actionability	no benefit	III	not actionable/unknown
**I744_P753delinsSNISG**	1 (0.11%)	Net loss of amino acids on exon 19 (deletion)	sensitive	IA	exon 19 deletion
**E746_A750del**	210 (22.7%)	Net loss of amino acids on exon 19 (deletion)	sensitive	IA	exon 19 deletion
**E746_A750del/G873E**	1 (0.11%)	Considered comparable to E746_A750del	sensitive	IA	exon 19 deletion
**E746_A750del/K754Q**	1 (0.11%)	Considered comparable to E746_A750del	sensitive	IA	exon 19 deletion
**E746_A750del/P848L**	1 (0.11%)	Considered comparable to E746_A750del	sensitive	IA	exon 19 deletion
**E746_A750del/V765M**	1 (0.11%)	Considered comparable to E746_A750del	sensitive	IA	exon 19 deletion
**E746_A750delinsEP**	1 (0.11%)	Net loss of amino acids on exon 19 (deletion)	sensitive	IA	exon 19 deletion
**E746_A750delinsIP**	1 (0.11%)	Net loss of amino acids on exon 19 (deletion)	sensitive	IA	exon 19 deletion
**E746_A750dup**	1 (0.11%)	Considered comparable to I740_K745dup	no benefit	III	not actionable/unknown
**E746_K754delinsVSR**	1 (0.11%)	Net loss of amino acids on exon 19 (deletion)	sensitive	IA	exon 19 deletion
**E746_L747delinsIP**	1 (0.11%)	Considered comparable to L747P	sensitive	IID	uncommon, actionable
**E746_P753delinsANKE**	1 (0.11%)	Net loss of amino acids on exon 19 (deletion)	sensitive	IA	exon 19 deletion
**E746_P753delinsIS**	1 (0.11%)	Net loss of amino acids on exon 19 (deletion)	sensitive	IA	exon 19 deletion
**E746_P753delinsVS**	2 (0.22%)	Net loss of amino acids on exon 19 (deletion)	sensitive	IA	exon 19 deletion
**E746_S752delinsI**	1 (0.11%)	Net loss of amino acids on exon 19 (deletion)	sensitive	IA	exon 19 deletion
**E746_S752delinsV**	20 (2.16%)	Net loss of amino acids on exon 19 (deletion)	sensitive	IA	exon 19 deletion
**E746_T751del**	2 (0.22%)	Net loss of amino acids on exon 19 (deletion)	sensitive	IA	exon 19 deletion
**E746_T751delinsA**	3 (0.32%)	Net loss of amino acids on exon 19 (deletion)	sensitive	IA	exon 19 deletion
**E746_T751delinsAA**	1 (0.11%)	Net loss of amino acids on exon 19 (deletion)	sensitive	IA	exon 19 deletion
**E746_T751delinsI**	2 (0.22%)	Net loss of amino acids on exon 19 (deletion)	sensitive	IA	exon 19 deletion
**E746_T751delinsK**	1 (0.11%)	Net loss of amino acids on exon 19 (deletion)	sensitive	IA	exon 19 deletion
**E746_T751delinsL**	1 (0.11%)	Net loss of amino acids on exon 19 (deletion)	sensitive	IA	exon 19 deletion
**E746_T751delinsP**	1 (0.11%)	Net loss of amino acids on exon 19 (deletion)	sensitive	IA	exon 19 deletion
**E746_T751delinsS**	1 (0.11%)	Net loss of amino acids on exon 19 (deletion)	sensitive	IA	exon 19 deletion
**E746_T751delinsVP**	1 (0.11%)	Net loss of amino acids on exon 19 (deletion)	sensitive	IA	exon 19 deletion
**L747_A750delinsP**	10 (1.08%)	Net loss of amino acids on exon 19 (deletion)	sensitive	IA	exon 19 deletion
**L747_A750delinsP/V845L**	1 (0.11%)	Considered comparable to L747_A750delinsP	sensitive	IA	exon 19 deletion
**L747_A755delinsSKD**	1 (0.11%)	Net loss of amino acids on exon 19 (deletion)	sensitive	IA	exon 19 deletion
**L747_E749del**	3 (0.32%)	Net loss of amino acids on exon 19 (deletion)	sensitive	IA	exon 19 deletion
**L747_K754delinsATSPE**	1 (0.11%)	Net loss of amino acids on exon 19 (deletion)	sensitive	IA	exon 19 deletion
**L747_K754delinsG**	1 (0.11%)	Net loss of amino acids on exon 19 (deletion)	sensitive	IA	exon 19 deletion
**L747_K754delinsQPN**	1 (0.11%)	Net loss of amino acids on exon 19 (deletion)	sensitive	IA	exon 19 deletion
**L747_P753delinsQ**	1 (0.11%)	Net loss of amino acids on exon 19 (deletion)	sensitive	IA	exon 19 deletion
**L747_P753delinsS**	25 (2.70%)	Net loss of amino acids on exon 19 (deletion)	sensitive	IA	exon 19 deletion
**L747_S752del**	7 (0.76%)	Net loss of amino acids on exon 19 (deletion)	sensitive	IA	exon 19 deletion
**L747_S752del/K754R**	1 (0.11%)	Considered comparable to L747_S752del	sensitive	IA	exon 19 deletion
**L747_S752del/L777Q**	1 (0.11%)	Considered comparable to L747_S752del	sensitive	IA	exon 19 deletion
**L747_S752delinsQ**	2 (0.22%)	Net loss of amino acids on exon 19 (deletion)	sensitive	IA	exon 19 deletion
**L747_T751del**	16 (1.73%)	Net loss of amino acids on exon 19 (deletion)	sensitive	IA	exon 19 deletion
**L747_T751del/S768I**	1 (0.11%)	Considered comparable to L747_T751del	sensitive	IA	exon 19 deletion
**L747_T751delinsP**	6 (0.65%)	Net loss of amino acids on exon 19 (deletion)	sensitive	IA	exon 19 deletion
**L747P**	4 (0.43%)	Clinical sensitivity to EGFR-TKI reported [70]	sensitive	IID	uncommon, actionable
**E749_A755delinsD**	1 (0.11%)	Net loss of amino acids on exon 19 (deletion)	sensitive	IA	exon 19 deletion
**A750_E758delinsP**	1 (0.11%)	Net loss of amino acids on exon 19 (deletion)	sensitive	IA	exon 19 deletion
**T751_I759delinsN**	1 (0.11%)	Net loss of amino acids on exon 19 (deletion)	sensitive	IA	exon 19 deletion
**S752_I759del**	4 (0.43%)	Net loss of amino acids on exon 19 (deletion)	sensitive	IA	exon 19 deletion
**P753L**	1 (0.11%)	No evidence on pathogenicity or actionability	no benefit	III	not actionable/unknown
**K757R**	1 (0.11%)	No evidence on pathogenicity or actionability	no benefit	III	not actionable/unknown
**A763S**	2 (0.22%)	No evidence on pathogenicity or actionability	no benefit	III	not actionable/unknown
**A763_Y764insFQEA**	1 (0.11%)	Net gain of amino acids on exon 20 (insertion)	no benefit	IIC	exon 20 insertion
**V765M**	2 (0.22%)	Only reported in combination with other variants, no evidence on individual variant	no benefit	III	not actionable/unknown
**A767_V769dup**	16 (1.73%)	Net gain of amino acids on exon 20 (insertion)	no benefit	IIC	exon 20 insertion
**A767T**	1 (0.11%)	No evidence on pathogenicity or actionability	no benefit	III	not actionable/unknown
**S768_D770dup**	9 (0.97%)	Net gain of amino acids on exon 20 (insertion)	no benefit	IIC	exon 20 insertion
**S768_V769delinsIL**	4 (0.43%)	Considered comparable to S768I	sensitive	IIC	uncommon, actionable
**S768I**	5 (0.54%)	Clinical sensitivity to EGFR-TKI reported [49]	sensitive	IIC	uncommon, actionable
**S768I/L861Q**	2 (0.22%)	Clinical sensitivity to EGFR-TKI reported [65]	sensitive	IIC	uncommon, actionable
**S768I/V774M**	2 (0.22%)	Considered comparable to S768I	sensitive	IIC	uncommon, actionable
**V769L**	2 (0.22%)	Only reported in combination with other variants, no evidence on individual variant	no benefit	III	not actionable/unknown
**V769M**	2 (0.22%)	Possible germline variant [71], no evidence sensitivity to EGFR-TKI	no benefit	III	not actionable/unknown
**D770_H773dup**	1 (0.11%)	Net gain of amino acids on exon 20 (insertion)	no benefit	IIC	exon 20 insertion
**D770_N771insG**	3 (0.32%)	Net gain of amino acids on exon 20 (insertion)	no benefit	IIC	exon 20 insertion
**D770_N771insGF**	1 (0.11%)	Net gain of amino acids on exon 20 (insertion)	no benefit	IIC	exon 20 insertion
**D770_N771insSVA**	2 (0.22%)	Net gain of amino acids on exon 20 (insertion)	no benefit	IIC	exon 20 insertion
**D770_N771insT**	1 (0.11%)	Net gain of amino acids on exon 20 (insertion)	no benefit	IIC	exon 20 insertion
**D770_N771insY**	1 (0.11%)	Net gain of amino acids on exon 20 (insertion)	no benefit	IIC	exon 20 insertion
**D770_P772dup**	1 (0.11%)	Net gain of amino acids on exon 20 (insertion)	no benefit	IIC	exon 20 insertion
**D770delinsEQPP**	1 (0.11%)	Net gain of amino acids on exon 20 (insertion)	no benefit	IIC	exon 20 insertion
**D770delinsGY**	3 (0.32%)	Net gain of amino acids on exon 20 (insertion)	no benefit	IIC	exon 20 insertion
**D770Y**	1 (0.11%)	No evidence on pathogenicity or actionability	no benefit	III	not actionable/unknown
**N771_H773dup**	2 (0.22%)	Net gain of amino acids on exon 20 (insertion)	no benefit	IIC	exon 20 insertion
**N771_P772insH**	1 (0.11%)	Net gain of amino acids on exon 20 (insertion)	no benefit	IIC	exon 20 insertion
**N771_P772insR**	1 (0.11%)	Net gain of amino acids on exon 20 (insertion)	no benefit	IIC	exon 20 insertion
**N771delinsGF**	1 (0.11%)	Net gain of amino acids on exon 20 (insertion)	no benefit	IIC	exon 20 insertion
**N771delinsGY**	1 (0.11%)	Net gain of amino acids on exon 20 (insertion)	no benefit	IIC	exon 20 insertion
**N771delinsKG**	1 (0.11%)	Net gain of amino acids on exon 20 (insertion)	no benefit	IIC	exon 20 insertion
**N771delinsTH**	1 (0.11%)	Net gain of amino acids on exon 20 (insertion)	no benefit	IIC	exon 20 insertion
**N771L**	1 (0.11%)	No evidence on pathogenicity or actionability	no benefit	III	not actionable/unknown
**P772_C775dup**	1 (0.11%)	Net gain of amino acids on exon 20 (insertion)	no benefit	IIC	exon 20 insertion
**P772_H773dup**	3 (0.32%)	Net gain of amino acids on exon 20 (insertion)	no benefit	IIC	exon 20 insertion
**P772_H773insANP**	1 (0.11%)	Net gain of amino acids on exon 20 (insertion)	no benefit	IIC	exon 20 insertion
**H773_V774dup**	2 (0.22%)	Net gain of amino acids on exon 20 (insertion)	no benefit	IIC	exon 20 insertion
**H773_V774insAH**	2 (0.22%)	Net gain of amino acids on exon 20 (insertion)	no benefit	IIC	exon 20 insertion
**H773delinsYNPY**	1 (0.11%)	Net gain of amino acids on exon 20 (insertion)	no benefit	IIC	exon 20 insertion
**H773dup**	5 (0.54%)	Net gain of amino acids on exon 20 (insertion)	no benefit	IIC	exon 20 insertion
**V774delinsHC**	1 (0.11%)	Net gain of amino acids on exon 20 (insertion)	no benefit	IIC	exon 20 insertion
**V774M**	3 (0.32%)	Only reported in combination with other variants, no evidence on individual variant	sensitive	III	not actionable/unknown
**V774M/L861R**	1 (0.11%)	Considered comparable to L861R	sensitive	IIC	uncommon, actionable
**C775F**	1 (0.11%)	No evidence on pathogenicity or actionability	no benefit	III	not actionable/unknown
**R776H**	1 (0.11%)	Only reported in combination with other variants, no evidence on individual variant	no benefit	III	not actionable/unknown
**R776L**	1 (0.11%)	No evidence on pathogenicity or actionability	no benefit	III	not actionable/unknown
**G779F**	3 (0.32%)	No evidence on pathogenicity or actionability	no benefit	III	not actionable/unknown
**G779S**	1 (0.11%)	No evidence on pathogenicity or actionability	no benefit	III	not actionable/unknown
**C781F**	1 (0.11%)	No evidence on pathogenicity or actionability	no benefit	III	not actionable/unknown
**Q787E**	1 (0.11%)	No evidence on pathogenicity or actionability	no benefit	III	not actionable/unknown
**T790M**	1 (0.11%)	Known resistance-inducing mutation, but not transforming without a driver mutationg [72]	no benefit	III	not actionable/unknown
**G796C**	1 (0.11%)	No evidence on pathogenicity or actionability	no benefit	III	not actionable/unknown
**L799M**	1 (0.11%)	No evidence on pathogenicity or actionability	no benefit	III	not actionable/unknown
**D830Y**	1 (0.11%)	No evidence on pathogenicity or actionability	no benefit	III	not actionable/unknown
**L832T**	1 (0.11%)	No evidence on pathogenicity or actionability	no benefit	III	not actionable/unknown
**L833V/H835L**	2 (0.22%)	Clinical sensitivity to EGFR-TKI reported [73]	sensitive	IID	uncommon, actionable
**R836L**	1 (0.11%)	No evidence on pathogenicity or actionability	no benefit	III	not actionable/unknown
**R836R**	4 (0.43%)	Likely a SNP due to high allele frequency in general population (1.7%; GnomAD) ^b^	no benefit	IV	not actionable/unknown
**A840T**	4 (0.43%)	No evidence on pathogenicity or actionability	no benefit	III	not actionable/unknown
**P848L**	5 (0.54%)	No evidence on pathogenicity or actionability, may be a rare SNP (AF 0.03; GnomAD) ^b^	no benefit	IV	not actionable/unknown
**L858_K860delinsRTI**	1 (0.11%)	Considered comparable to L858R	sensitive	IID	L858R
**L858R**	274 (29.6%)	Classical, well known activating mutation sensitive to first- and second-generation EGFR-TKI	sensitive	IA	L858R
**L858R/A871E**	1 (0.11%)	Considered comparable to L858R	sensitive	IA	L858R
**L858R/A871G**	1 (0.11%)	Considered comparable to L858R	sensitive	IA	L858R
**L858R/E709G**	1 (0.11%)	Considered comparable to L858R	sensitive	IA	L858R
**L858R/L718M**	1 (0.11%)	Considered comparable to L858R	sensitive	IA	L858R
**L858R/L747V**	1 (0.11%)	Considered comparable to L858R	sensitive	IA	L858R
**L858R/R776H**	2 (0.22%)	Considered comparable to L858R	sensitive	IA	L858R
**L858R/S768I**	4 (0.43%)	Considered comparable to L858R	sensitive	IA	L858R
**L858R/V834L**	1 (0.11%)	Considered comparable to L858R	sensitive	IA	L858R
**A859T**	1 (0.11%)	Clinical sensitivity to EGFR-TKI reported [74]	sensitive	IID	uncommon, actionable
**L861Q**	19 (2.05%)	Clinical sensitivity to EGFR-TKI reported [75]	sensitive	IIC	uncommon, actionable
**L861R**	3 (0.32%)	Clinical sensitivity to EGFR-TKI reported [75]	sensitive	IIC	uncommon, actionable
**G863D**	1 (0.11%)	No evidence on pathogenicity or actionability	no benefit	III	not actionable/unknown
**A864T**	1 (0.11%)	No evidence on pathogenicity or actionability	no benefit	III	not actionable/unknown
**A864V**	1 (0.11%)	No evidence on pathogenicity or actionability	no benefit	III	not actionable/unknown
**E866K**	1 (0.11%)	No evidence on pathogenicity or actionability	no benefit	III	not actionable/unknown
***EGFR* variant, NOS**	4 (0.43%)	Exact variant and exon not reported	unknown	NA	unspecified
**Exon 18 variant, NOS + Exon 20 variant, NOS**	2 (0.22%)	Exact variant not reported	unknown	NA	unspecified
**Exon 19 deletion, NOS**	52 (5.62%)	Exact variant not reported but confirmed net loss of amino acids on exon 19 (deletion)	unknown	NA	exon 19 deletion
**Exon 19 variant, NOS**	3 (0.32%)	Exact variant not reported (could be a deletion or a single-nucleotide variant)	sensitive	IA	unspecified
**Exon 20 insertion, NOS**	3 (0.32%)	Exact variant not reported but confirmed net gain of amino acids on exon 21 (insertion)	unknown	NA	exon 20 insertion
**Exon 20 SNV (silent)**	1 (0.11%)	Silent mutation, no change in amino acids	no benefit	IIC	not actionable/unknown
**Exon 21 variant, NOS**	3 (0.32%)	Exact variant not reported (could be L858R or a different, non-classical mutation)	no benefit	IV	unspecified

^a^ Amount of tumors reported to harbor this mutation, with percentage of all *EGFR*-mutant tumors between brackets. ^b^ According to GnomAD [76], query on 18 December 2020. Abbreviations: AF, allele frequency; DOI, digital object identifier; *EGFR*, epidermal growth factor receptor; LoE, AMP/ASCO/CAP Level of evidence for actionability [20]; NOS, not otherwise specified; NSCLC, non-small cell lung cancer; SNP, single nucleotide polymorphism; TKI, tyrosine kinase inhibitor.

## Data Availability

The data presented in this study are available on request from the corresponding author.

## Supplementary Materials

The following are available online at https://www.mdpi.com/article/10.3390/cancers13143641/s1, Figure S1: Inter-pathology department variance in *EGFR* testing request rates, Figure S2: Changes in percentage of patients treated with targeted therapy in first-line of treatment.

## References

[1] Bray F., Ferlay J., Soerjomataram I., Siegel R.L., Torre L.A., Jemal A. (2018). Global cancer statistics 2018: GLOBOCAN estimates of incidence and mortality worldwide for 36 cancers in 185 countries. CA Cancer J. Clin..

[2] (2017). AACR Project GENIE Consortium AACR Project GENIE: Powering precision medicine through an international consortium. Cancer Discov..

[3] Sequist L.V., Martins R.G., Spigel D., Grunberg S.M., Spira A., Jänne P.A., Joshi V.A., McCollum D., Evans T.L., Muzikansky A. (2008). First-line gefitinib in patients with advanced non-small-cell lung cancer harboring somatic EGFR mutations. J. Clin. Oncol..

[4] Wu Y.-L., Zhou C., Liam C.-K., Wu G., Liu X., Zhong Z., Lu S., Cheng Y., Han B., Chen L. (2015). First-line erlotinib versus gemcitabine/cisplatin in patients with advanced EGFR mutation-positive non-small-cell lung cancer: Analyses from the phase III, randomized, open-label, ENSURE study. Ann. Oncol..

[5] Park K., Tan E.-H., O’Byrne K., Zhang L., Boyer M., Mok T., Hirsh V., Yang J.C.-H., Lee K.H., Lu S. (2016). Afatinib versus gefitinib as first-line treatment of patients with EGFR mutation-positive non-small-cell lung cancer (LUX-Lung 7): A phase 2B, open-label, randomised controlled trial. Lancet Oncol..

[6] Wu Y.-L., Cheng Y., Zhou X., Lee K.H., Nakagawa K., Niho S., Tsuji F., Linke R., Rosell R., Corral J. (2017). Dacomitinib versus gefitinib as first-line treatment for patients with EGFR-mutation-positive non-small-cell lung cancer (ARCHER 1050): A randomised, open-label, phase 3 trial. Lancet Oncol..

[7] Soria J.-C., Ohe Y., Vansteenkiste J., Reungwetwattana T., Chewaskulyong B., Lee K.H., Dechaphunkul A., Imamura F., Nogami N., Kurata T. (2018). Osimertinib in untreated EGFR-mutated advanced non-small-cell lung cancer. N. Engl. J. Med..

[8] D’Addario G., Früh M., Reck M., Baumann P., Klepetko W., Felip E., ESMO Guidelines Working Group (2010). Metastatic non-small-cell lung cancer: ESMO Clinical Practice Guidelines for diagnosis, treatment and follow-up. Ann. Oncol..

[9] Planchard D., Popat S., Kerr K., Novello S., Smit E.F., Faivre-Finn C., Mok T.S., Reck M., van Schil P.E., Hellmann M.D. (2018). Metastatic non-small cell lung cancer: ESMO Clinical Practice Guidelines for diagnosis, treatment and follow-up. Ann. Oncol..

[10] Hendriks L.E.L., Dingemans A.-M.C., de Ruysscher D.K.M., Aarts M.J., Barberio L., Cornelissen R., Hartemink K.J., van den Heuvel M., Schuuring E., Smit H.J.M. (2021). Lung cancer in the Netherlands. J. Thorac. Oncol..

[11] Goodwin S., McPherson J.D., McCombie W.R. (2016). Coming of age: Ten years of next-generation sequencing technologies. Nat. Rev. Genet..

[12] Lin M.-T., Mosier S.L., Thiess M., Beierl K.F., Debeljak M., Tseng L.-H., Chen G., Yegnasubramanian S., Ho H., Cope L. (2014). Clinical validation of KRAS, BRAF, and EGFR mutation detection using next-generation sequencing. Am. J. Clin. Pathol..

[13] Planchard D., Besse B., Groen H.J.M., Souquet P.-J., Quoix E., Baik C.S., Barlesi F., Kim T.M., Mazieres J., Novello S. (2016). Dabrafenib plus trametinib in patients with previously treated BRAF(V600E)-mutant metastatic non-small cell lung cancer: An open-label, multicentre phase 2 trial. Lancet Oncol..

[14] Wolf J., Seto T., Han J.-Y., Reguart N., Garon E.B., Groen H.J.M., Tan D.S.W., Hida T., de Jonge M., Orlov S.V. (2020). Capmatinib in MET Exon 14-mutated or MET-amplified non-small-cell lung cancer. N. Engl. J. Med..

[15] Li B.T., Shen R., Buonocore D., Olah Z.T., Ni A., Ginsberg M.S., Ulaner G.A., Offin M., Feldman D., Hembrough T. (2018). Ado-trastuzumab emtansine for patients with HER2-mutant lung cancers: Results from a phase II basket trial. J. Clin. Oncol..

[16] UMCG Pathologie en Medische Biologie Moleculaire Diagnostiek. https://www.umcg.nl/NL/UMCG/Afdelingen/Pathologie/Professionals/moleculaire-diagnostiek/Paginas/default.aspx.

[17] Casparie M., Tiebosch A.T.M.G., Burger G., Blauwgeers H., van de Pol A., van Krieken J.H.J.M., Meijer G.A. (2007). Pathology databanking and biobanking in The Netherlands, a central role for PALGA, the nationwide histopathology and cytopathology data network and archive. Cell. Oncol..

[18] NVALT Niet-Kleincellig Longcarcinoom: Landelijke Richtlijn. https://richtlijnendatabase.nl/richtlijn/niet_kleincellig_longcarcinoom/startpagina_-_niet-kleincelling_longcarcinoom.html.

[19] Den Dunnen J.T., Dalgleish R., Maglott D.R., Hart R.K., Greenblatt M.S., McGowan-Jordan J., Roux A.-F., Smith T., Antonarakis S.E., Taschner P.E.M. (2016). HGVS Recommendations for the description of sequence variants: 2016 update. Hum. Mutat..

[20] Li M.M., Datto M., Duncavage E.J., Kulkarni S., Lindeman N.I., Roy S., Tsimberidou A.M., Vnencak-Jones C.L., Wolff D.J., Younes A. (2017). Standards and guidelines for the interpretation and reporting of sequence variants in cancer: A joint consensus recommendation of the Association for Molecular Pathology, American Society of Clinical Oncology, and College of American Pathologists. J. Mol. Diagn..

[21] Heigener D.F., Schumann C., Sebastian M., Sadjadian P., Stehle I., Märten A., Lüers A., Griesinger F., Scheffler M., Abdollahi A. (2015). Afatinib in non-small cell lung cancer harboring uncommon EGFR mutations pretreated with reversible EGFR inhibitors. Oncologist.

[22] Deans Z.C., Costa J.L., Cree I., Dequeker E., Edsjö A., Henderson S., Hummel M., Ligtenberg M.J., Loddo M., Machado J.C. (2017). Integration of next-generation sequencing in clinical diagnostic molecular pathology laboratories for analysis of solid tumours; an expert opinion on behalf of IQN Path ASBL. Virchows Arch..

[23] Boonstra P.A., ter Elst A., Tibbesma M., Bosman L.J., Mathijssen R., Atrafi F., van Coevorden F., Steeghs N., Farag S., Gelderblom H. (2018). A single digital droplet PCR assay to detect multiple KIT exon 11 mutations in tumor and plasma from patients with gastrointestinal stromal tumors. Oncotarget.

[24] Sie D., Snijders P.J.F., Meijer G.A., Doeleman M.W., van Moorsel M.I.H., van Essen H.F., Eijk P.P., Grünberg K., van Grieken N.C.T., Thunnissen E. (2014). Performance of amplicon-based next generation DNA sequencing for diagnostic gene mutation profiling in oncopathology. Cell. Oncol..

[25] Steeghs E.M.P., Kroeze L.I., Tops B.B.J., van Kempen L.C., ter Elst A., Kastner-van Raaij A.W.M., Hendriks-Cornelissen S.J.B., Hermsen M.J.W., Jansen E.A.M., Nederlof P.M. (2020). Comprehensive routine diagnostic screening to identify predictive mutations, gene amplifications, and microsatellite instability in FFPE tumor material. BMC Cancer.

[26] Gijtenbeek R.G.P., Damhuis R.A.M., Groen H.J.M., van der Wekken A.J., van Geffen W.H. (2020). Nationwide real-world cohort study of first-line tyrosine kinase inhibitor treatment in epidermal growth factor receptor-mutated non-small-cell lung cancer. Clin. Lung Cancer.

[27] Peters S., Adjei A.A., Gridelli C., Reck M., Kerr K., Felip E., ESMO Guidelines Working Group (2012). Metastatic non-small-cell lung cancer (NSCLC): ESMO Clinical Practice Guidelines for diagnosis, treatment and follow-up. Ann. Oncol..

[28] Smits A.J.J., Kummer J.A., Hinrichs J.W.J., Herder G.J.M., Scheidel-Jacobse K.C., Jiwa N.M., Ruijter T.E.G., Nooijen P.T.G.A., Looijen-Salamon M.G., Ligtenberg M.J.L. (2012). EGFR and KRAS mutations in lung carcinomas in the Dutch population: Increased EGFR mutation frequency in malignant pleural effusion of lung adenocarcinoma. Cell. Oncol..

[29] Zhang Y.-L., Yuan J.-Q., Wang K.-F., Fu X.-H., Han X.-R., Threapleton D., Yang Z.-Y., Mao C., Tang J.-L. (2016). The prevalence of EGFR mutation in patients with non-small cell lung cancer: A systematic review and meta-analysis. Oncotarget.

[30] Brindel A., Althakfi W., Barritault M., Bringuier P.-P., Watkin E., Maury J.-M., Girard N., Brevet M. (2018). Uncommon EGFR mutations in lung adenocarcinomas: Clinical features and response to tyrosine kinase inhibitors. Ann. Oncol..

[31] Leduc C., Merlio J.P., Besse B., Blons H., Debieuvre D., Bringuier P.P., Monnet I., Rouquette I., Fraboulet-Moreau S., Lemoine A. (2017). Clinical and molecular characteristics of non-small-cell lung cancer (NSCLC) harboring EGFR mutation: Results of the nationwide French Cooperative Thoracic Intergroup (IFCT) program. Ann. Oncol..

[32] Yun C.-H., Mengwasser K.E., Toms A.V., Woo M.S., Greulich H., Wong K.-K., Meyerson M., Eck M.J. (2008). The T790M mutation in EGFR kinase causes drug resistance by increasing the affinity for ATP. Proc. Natl. Acad. Sci. USA.

[33] Cross D.A.E., Ashton S.E., Ghiorghiu S., Eberlein C., Nebhan C.A., Spitzler P.J., Orme J.P., Finlay M.R.V., Ward R.A., Mellor M.J. (2014). AZD9291, an irreversible EGFR TKI, overcomes T790M-mediated resistance to EGFR inhibitors in lung cancer. Cancer Discov..

[34] Reck M., Rodríguez-Abreu D., Robinson A.G., Hui R., Csőszi T., Fülöp A., Gottfried M., Peled N., Tafreshi A., Cuffe S. (2016). Pembrolizumab versus chemotherapy for PD-L1-positive non-small-cell lung cancer. N. Engl. J. Med..

[35] Lee C.K., Man J., Lord S., Links M., Gebski V., Mok T., Yang J.C.-H. (2017). Checkpoint inhibitors in metastatic EGFR-mutated non-small cell lung cancer—A meta-analysis. J. Thorac. Oncol..

[36] Kwak E.L., Bang Y.-J., Camidge D.R., Shaw A.T., Solomon B., Maki R.G., Ou S.-H.I., Dezube B.J., Jänne P.A., Costa D.B. (2010). Anaplastic lymphoma kinase inhibition in non-small-cell lung cancer. N. Engl. J. Med..

[37] Drilon A., Siena S., Ou S.-H.I., Patel M., Ahn M.J., Lee J., Bauer T.M., Farago A.F., Wheler J.J., Liu S.V. (2017). Safety and antitumor activity of the multitargeted pan-TRK, ROS1, and ALK inhibitor entrectinib: Combined results from two phase I trials (ALKA-372-001 and STARTRK-1). Cancer Discov..

[38] Drilon A., Oxnard G.R., Tan D.S.W., Loong H.H.F., Johnson M., Gainor J., McCoach C.E., Gautschi O., Besse B., Cho B.C. (2020). Efficacy of selpercatinib in RET fusion-positive non-small-cell lung cancer. N. Engl. J. Med..

[39] Shaw A.T., Riely G.J., Bang Y.-J., Kim D.-W., Camidge D.R., Solomon B.J., Varella-Garcia M., Iafrate A.J., Shapiro G.I., Usari T. (2019). Crizotinib in ROS1-rearranged advanced non-small-cell lung cancer (NSCLC): Updated results, including overall survival, from PROFILE 1001. Ann. Oncol..

[40] Burns T.F., Borghaei H., Ramalingam S.S., Mok T.S., Peters S. (2020). Targeting KRAS-mutant non-small-cell lung cancer: One mutation at a time, with a focus on KRAS G12C mutations. J. Clin. Oncol..

[41] Gristina V., la Mantia M., Galvano A., Cutaia S., Barraco N., Castiglia M., Perez A., Bono M., Iacono F., Greco M. (2021). Non-small cell lung cancer harboring concurrent EGFR genomic alterations: A systematic review and critical appraisal of the double dilemma. J. Mol. Pathol..

[42] Canale M., Petracci E., Delmonte A., Chiadini E., Dazzi C., Papi M., Capelli L., Casanova C., de Luigi N., Mariotti M. (2017). Impact of TP53 mutations on outcome in EGFR-mutated patients treated with first-line tyrosine kinase inhibitors. Clin. Cancer Res..

[43] Horinouchi H. (2020). To combine or not to combine: Anti-vascular endothelial growth factor therapies in EGFR mutation positive non-small cell lung cancer. Ann. Transl. Med..

[44] Nakagawa K., Garon E.B., Seto T., Nishio M., Ponce Aix S., Paz-Ares L., Chiu C.-H., Park K., Novello S., Nadal E. (2019). Ramucirumab plus erlotinib in patients with untreated, EGFR-mutated, advanced non-small-cell lung cancer (RELAY): A randomised, double-blind, placebo-controlled, phase 3 trial. Lancet Oncol..

[45] Zhao H., Yao W., Min X., Gu K., Yu G., Zhang Z., Cui J., Miao L., Zhang L., Yuan X. (2021). Apatinib plus gefitinib as first-line treatment in advanced EGFR-mutant NSCLC: The phase III ACTIVE study (CTONG1706). J. Thorac. Oncol..

[46] Suda K., Mitsudomi T., Shintani Y., Okami J., Ito H., Ohtsuka T., Toyooka S., Mori T., Watanabe S.-I., Asamura H. (2021). Clinical impacts of EGFR mutation status: Analysis of 5780 surgically resected lung cancer cases. Ann. Thorac. Surg..

[47] Bergqvist M., Christensen H.N., Wiklund F., Bergström S. (2020). Real world utilization of EGFR TKIs and prognostic factors for survival in NSCLC during 2010–2016 in Sweden: A nationwide observational study. Int. J. Cancer.

[48] Aye P.S., McKeage M.J., Tin Tin S., Khwaounjoo P., Elwood J.M. (2020). Factors associated with overall survival in a population-based cohort of non- squamous NSCLC patients from northern New Zealand: A comparative analysis by EGFR mutation status. Cancer Epidemiol..

[49] Yang J.C.-H., Sequist L.V., Geater S.L., Tsai C.-M., Mok T.S.K., Schuler M., Yamamoto N., Yu C.-J., Ou S.-H.I., Zhou C. (2015). Clinical activity of afatinib in patients with advanced non-small-cell lung cancer harbouring uncommon EGFR mutations: A combined post-hoc analysis of LUX-Lung 2, LUX-Lung 3, and LUX-Lung 6. Lancet Oncol..

[50] Passaro A., Prelaj A., Bonanno L., Tiseo M., Tuzi A., Proto C., Chiari R., Rocco D., Genova C., Sini C. (2019). Activity of EGFR TKIs in Caucasian patients with NSCLC harboring potentially sensitive uncommon EGFR mutations. Clin. Lung Cancer.

[51] Pilotto S., Rossi A., Vavalà T., Follador A., Tiseo M., Galetta D., Morabito A., di Maio M., Martelli O., Caffo O. (2018). Outcomes of first-generation EGFR-TKIs against non-small-cell lung cancer harboring uncommon EGFR mutations: A post hoc analysis of the BE-POSITIVE study. Clin. Lung Cancer.

[52] Yasuda H., Park E., Yun C.-H., Sng N.J., Lucena-Araujo A.R., Yeo W.-L., Huberman M.S., Cohen D.W., Nakayama S., Ishioka K. (2013). Structural, biochemical, and clinical characterization of epidermal growth factor receptor (EGFR) exon 20 insertion mutations in lung cancer. Sci. Transl. Med..

[53] Arrieta O., Cardona A.F., Corrales L., Campos-Parra A.D., Sánchez-Reyes R., Amieva-Rivera E., Rodríguez J., Vargas C., Carranza H., Otero J. (2015). The impact of common and rare EGFR mutations in response to EGFR tyrosine kinase inhibitors and platinum-based chemotherapy in patients with non-small cell lung cancer. Lung Cancer.

[54] Chang L.-C., Lim C.-K., Chang L.-Y., Chen K.-Y., Shih J.-Y., Yu C.-J. (2019). Non-small cell lung cancer harbouring non-resistant uncommon EGFR mutations: Mutation patterns, effectiveness of epidermal growth factor receptor-tyrosine kinase inhibitors and prognostic factors. Eur. J. Cancer.

[55] Shi J., Yang H., Jiang T., Li X., Zhao C., Zhang L., Zhao S., Liu X., Jia Y., Wang Y. (2017). Uncommon EGFR mutations in a cohort of Chinese NSCLC patients and outcomes of first-line EGFR-TKIs and platinum-based chemotherapy. Chin. J. Cancer Res..

[56] Jackman D.M., Yeap B.Y., Sequist L.V., Lindeman N., Holmes A.J., Joshi V.A., Bell D.W., Huberman M.S., Halmos B., Rabin M.S. (2006). Exon 19 deletion mutations of epidermal growth factor receptor are associated with prolonged survival in non-small cell lung cancer patients treated with gefitinib or erlotinib. Clin. Cancer Res..

[57] IASCLC Deeper Understanding of EGFR Mutation Subgroups Will Further Personalized Treatment for NSCLC. https://www.iaslc.org/iaslc-news/ilcn/deeper-understanding-egfr-mutation-subgroups-will-further-personalize-treatment.

[58] Gristina V., Malapelle U., Galvano A., Pisapia P., Pepe F., Rolfo C., Tortorici S., Bazan V., Troncone G., Russo A. (2020). The significance of epidermal growth factor receptor uncommon mutations in non-small cell lung cancer: A systematic review and critical appraisal. Cancer Treat. Rev..

[59] Lee J.C., Vivanco I., Beroukhim R., Huang J.H.Y., Feng W.L., DeBiasi R.M., Yoshimoto K., King J.C., Nghiemphu P., Yuza Y. (2006). Epidermal growth factor receptor activation in glioblastoma through novel missense mutations in the extracellular domain. PLoS Med..

[60] Ackerman A., Goldstein M.A., Kobayashi S., Costa D.B. (2012). EGFR delE709_T710insD: A rare but potentially EGFR inhibitor responsive mutation in non–small-cell lung cancer. J. Thorac. Oncol..

[61] Koopman B., van der Wekken A.J., ter Elst A., Hiltermann T.J.N., Vilacha J.F., Groves M.R., van den Berg A., Hiddinga B.I., Hijmering-Kappelle L.B.M., Stigt J.A. (2020). Relevance and effectiveness of molecular tumor board recommendations for patients with non–small-cell lung cancer with rare or complex mutational profiles. JCO Precis. Oncol..

[62] Cappuzzo F., Soo R., Hochmair M., Schuler M., Lam K.C., Stehle G., Cseh A., Lorence R.M., Linden S., Forman N.D. (2018). Global named patient use program of afatinib in advanced non-small-cell lung carcinoma patients who progressed following prior therapies. Future Oncol..

[63] Sehgal K., Rangachari D., VanderLaan P.A., Kobayashi S.S., Costa D.B. (2021). clinical benefit of tyrosine kinase inhibitors in advanced lung cancer with EGFR -G719A and other uncommon EGFR mutations. Oncologist.

[64] Tanaka I., Morise M., Kodama Y., Matsui A., Ozawa N., Ozone S., Goto D., Miyazawa A., Hase T., Hashimoto N. (2019). Potential for afatinib as an optimal treatment for advanced non-small cell lung carcinoma in patients with uncommon EGFR mutations. Lung Cancer.

[65] Liang S.-K., Hsieh M.-S., Lee M.-R., Keng L.-T., Ko J.-C., Shih J.-Y. (2017). Real-world experience of afatinib as a first-line therapy for advanced EGFR mutation-positive lung adenocarcinoma. Oncotarget.

[66] Brueckl W.M., Reck M., Schäfer H., Kortsik C., Gaska T., Rawluk J., Krüger S., Kokowski K., Budweiser S., Hoffmann C. (2020). Efficacy of afatinib in the clinical practice: Final results of the GIDEON study in EGFR mutated non-small cell lung cancer (NSCLC) in Germany. J. Clin. Oncol..

[67] Caliman E., Petreni P., Brugia M., Antonuzzo L., Mazzoni F. (2020). In regard to “Activity of EGFR TKIs in Caucasian Patients with NSCLC harbouring potentially sensitive uncommon EGFR mutations”. Clin. Lung Cancer.

[68] Zhang C., Lin L., Zuo R., Wang Y., Chen P. (2020). Response to tyrosine kinase inhibitors in lung adenocarcinoma with the rare epidermal growth factor receptor mutation S768I and G724S: A case report and literature review. Thorac. Cancer.

[69] Xu J., Jiang Q., Xu H., Liu A., Huang L. (2020). Two patients having NSCLC with novel duplication mutation in their EGFR gene (p.I740_K745dupIPVAIK) and their response to osimertinib. J. Thorac. Oncol..

[70] Liang S.-K., Ko J.-C., Yang J.C.-H., Shih J.-Y. (2019). Afatinib is effective in the treatment of lung adenocarcinoma with uncommon EGFR p.L747P and p.L747S mutations. Lung Cancer.

[71] Hellmann M.D., Hayashi T., Reva B., Yu H.A., Riely G.J., Adusumilli P.S., Travis W.D., Wilkins O., Bramletta N., Chandramohan R. (2017). Identification and functional characterization of EGFR V769M, a novel germline variant associated with multiple lung adenocarcinomas. JCO Precis. Oncol..

[72] Huo R., Li J., Li X., Shi J., Wang K., Jiao J., Shang Y. (2020). Significant benefits of osimertinib against adenosquamous carcinoma harboring germline T790M mutation. Oncologist.

[73] Long X., Qin T., Lin J. (2020). Great efficacy of afatinib in a patient with lung adenocarcinoma harboring EGFR L833V/H835L mutations: A case report. OncoTargets Ther..

[74] Le Maignan L., Mirebeau-Prunier D., Vervueren L., Jeanfaivre T., Urban T., Hureaux J. (2011). First case of A859T epidermal growth factor receptor mutation responding to erlotinib. J. Thorac. Oncol..

[75] Wu J.-Y., Yu C.-J., Chang Y.-C., Yang C.-H., Shih J.-Y., Yang P.-C. (2011). Effectiveness of Tyrosine kinase inhibitors on “uncommon” epidermal growth factor receptor mutations of unknown clinical significance in non–small cell lung cancer. Clin. Cancer Res..

[76] Karczewski K.J., Francioli L.C., Tiao G., Cummings B.B., Alföldi J., Wang Q., Collins R.L., Laricchia K.M., Ganna A., Birnbaum D.P. (2020). The mutational constraint spectrum quantified from variation in 141,456 humans. Nature.

[77] Cho J.H., Lim S.H., An H.J., Kim K.H., Park K.U., Kang E.J., Choi Y.H., Ahn M.S., Lee M.H., Sun J.-M. (2020). Osimertinib for patients with non-small-cell lung cancer harboring uncommon EGFR mutations: A multicenter, open-label, phase II trial (KCSG-LU15-09). J. Clin. Oncol..

[78] Yasuda H., Kobayashi S., Costa D.B. (2012). EGFR exon 20 insertion mutations in non-small-cell lung cancer: Preclinical data and clinical implications. Lancet Oncol..

[79] Yun J., Lee S.-H., Kim S.-Y., Jeong S.-Y., Kim J.-H., Pyo K.-H., Park C.-W., Heo S.G., Yun M.R., Lim S. (2020). Antitumor activity of amivantamab (JNJ-61186372), an EGFR-MET bispecific antibody, in diverse models of EGFR exon 20 insertion-driven NSCLC. Cancer Discov..

[80] Robichaux J.P., Elamin Y.Y., Tan Z., Carter B.W., Zhang S., Liu S., Li S., Chen T., Poteete A., Estrada-Bernal A. (2018). Mechanisms and clinical activity of an EGFR and HER2 exon 20-selective kinase inhibitor in non-small cell lung cancer. Nat. Med..

[81] Riely G.J., Neal J.W., Camidge D.R., Spira A.I., Piotrowska Z., Costa D.B., Tsao A.S., Patel J.D., Gadgeel S.M., Bazhenova L. (2021). Activity and safety of mobocertinib (TAK-788) in previously treated non-small cell lung cancer with EGFR exon 20 insertion mutations from a phase 1/2 trial. Cancer Discov..

[82] Hasegawa H., Yasuda H., Hamamoto J., Masuzawa K., Tani T., Nukaga S., Hirano T., Kobayashi K., Manabe T., Terai H. (2019). Efficacy of afatinib or osimertinib plus cetuximab combination therapy for non-small-cell lung cancer with EGFR exon 20 insertion mutations. Lung Cancer.

[83] Koopman B., Groen H.J.M., Ligtenberg M.J.L., Grünberg K., Monkhorst K., Langen A.J., Boelens M.C., Paats M.S., Thüsen J.H., Dinjens W.N.M. (2020). Multicenter comparison of molecular tumor boards in The Netherlands: Definition, composition, methods, and targeted therapy recommendations. Oncologist.

