# Supplementary material for: A Nationwide Study on the Impact of Routine Testing for EGFR Mutations in Advanced NSCLC Reveals Distinct Survival Patterns Based on EGFR Mutation Subclasses
Source: Cancers (Basel). 2021 Jul 20;13(14):3641. doi: 10.3390/cancers13143641 (PMC8307492; doi:10.3390/cancers13143641)
Supplement: Supplementary file 1 [file cancers-13-03641-s001.zip › cancers-1313144-supplementary.pdf]

A Nationwide Study on the Impact of Routine Testing for *EGFR* Mutations in Advanced NSCLC Reveals Distinct Survival Patterns Based on *EGFR* Mutation Subclasses

Bart Koopman, Betzabel N. Cajiao Garcia, Chantal C. H. J. Kuijpers, Ronald A. M. Damhuis, Anthonie J. van der Wekken, Harry J. M. Groen, Ed Schuuring, Stefan M. Willems and Léon C. van Kempen

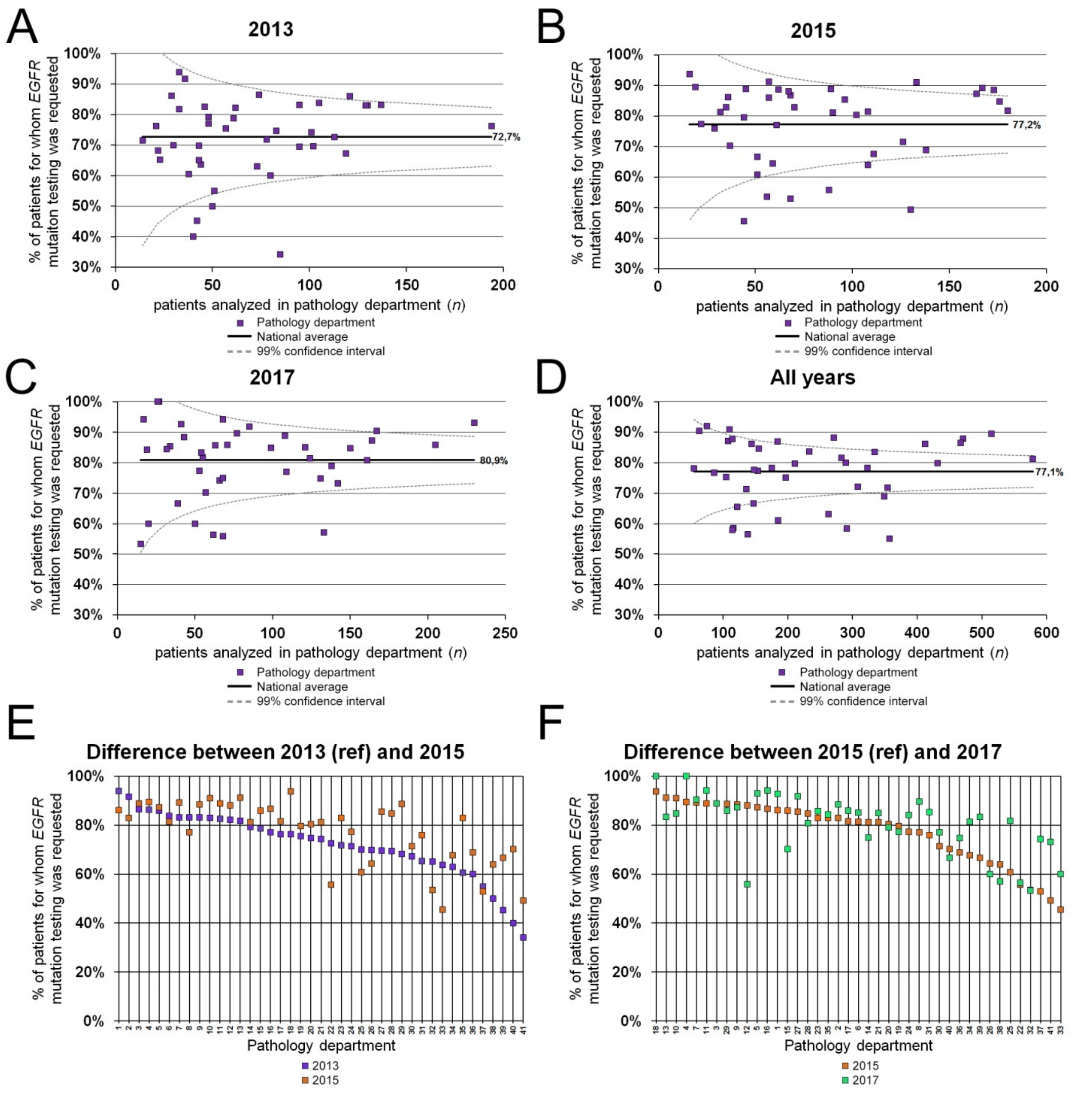

Figure S1. Inter-pathology department differences in *EGFR* test requests. Samples from the patients in this study originated from 52 different pathology departments throughout the Netherlands. Forty-one departments requested *EGFR* mutation testing for 10 or more patients in each of the three study years (2013, 2015 and 2017). It should be emphasized that the number of pathology departments is not the same as the number of laboratories that perform *EGFR* testing. The majority of non-academic pathology departments outsource molecular diagnostics to central (often academic) molecular pathology laboratories. (**A**–**D**) Volume of advanced non-squamous NSCLC (*x*-axis) by *EGFR* test request rate (*y*-axis) for

each individual department (purple squares), with 99% confidence intervals (dashed line) and national average (solid line), in 2013 (**A**), 2015 (**B**), 2017 (**C**) and all years combined (**D**). The variance in percentage of patients for whom *EGFR* testing was requested by a pathology department gradually decreased, from 34.1–93.9% in 2013 (**A**) and 45.5–93.8% in 2015 (**B**) to 53.3–100% in 2017 (**C**). Although these variances suggest underperformance by some departments, most were within the 99.7% CI based on the national average and their respective tumor volumes. In 2017, statistical underperformance was found in only four out of the 41 departments (9.8%) (**C**). In other words, these departments requested fewer *EGFR* mutation tests (<60% of the eligible patients) than expected for their respective tumor volumes and the national average of 80.9% in 2017. (**E,F**) Differences in testing rates (*y*-axis) by department (*x*-axis) in 2013 (purple) versus 2015 (orange) (**E**) and 2015 (orange) versus 2017 (green) (**F**). The majority of departments improved their request for *EGFR* testing from 2013 to 2015 (31/41; 76%; **E**) and 2015 to 2017 (27/41; 66%; **F**).

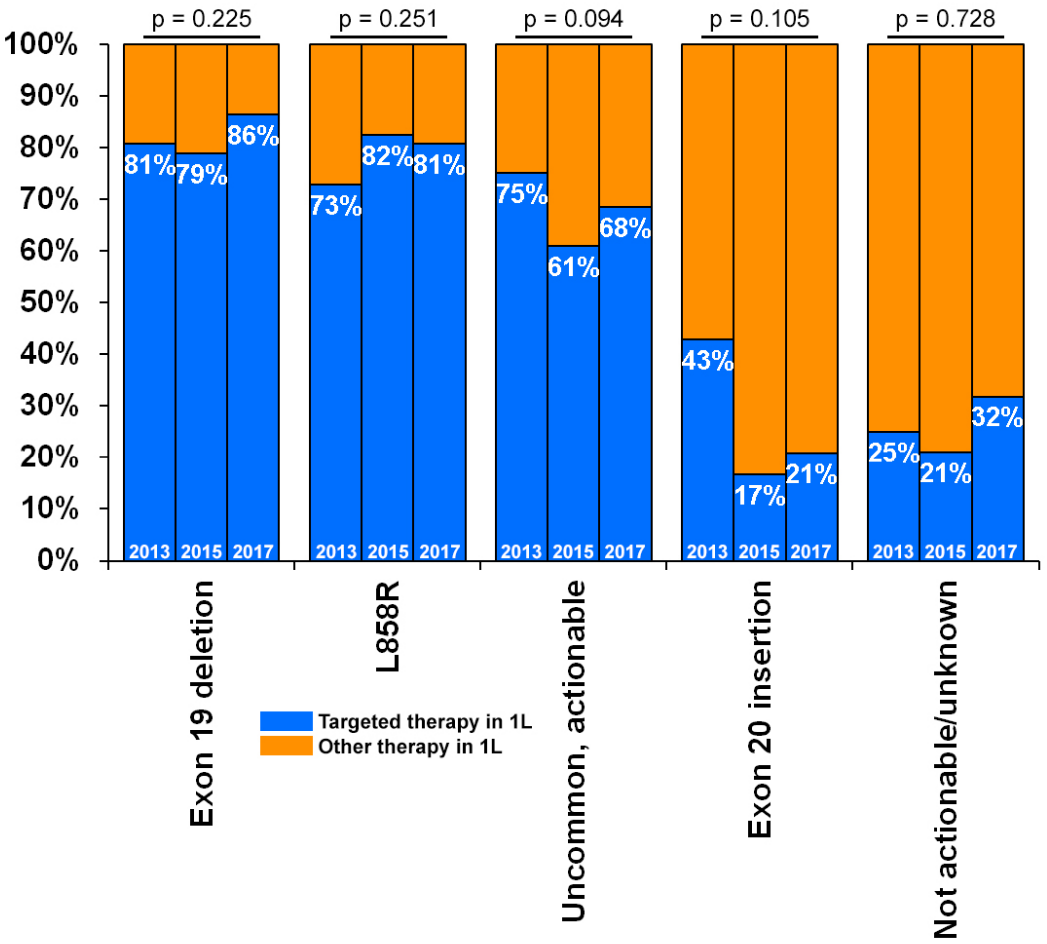

Figure S2. Changes in percentage of patients treated with targeted therapy in first line of treatment. Description: Clustered bar chart depicting the percentage of patients treated with first-line targeted therapy (blue) versus other therapy (orange). Level of significance was assessed with Pearson's Chi-square test. There were no significant differences between the three years. Abbreviations: 1L, first line; p, level of significance.
